# Resolvin D1 prevents epithelial-mesenchymal transition and reduces the stemness features of hepatocellular carcinoma by inhibiting paracrine of cancer-associated fibroblast-derived COMP

**DOI:** 10.1186/s13046-019-1163-6

**Published:** 2019-04-18

**Authors:** Liankang Sun, Yufeng Wang, Liang Wang, Bowen Yao, Tianxiang Chen, Qing Li, Zhikui Liu, Runkun Liu, Yongshen Niu, Tao Song, Qingguang Liu, Kangsheng Tu

**Affiliations:** Department of Hepatobiliary Surgery, The First Affiliated Hospital of Xi’an Jiaotong University, 277 Yanta West Road, Xi’an, Shaanxi Province 710061 China

**Keywords:** Hepatocellular carcinoma, Cancer-associated fibroblasts, COMP, Resolvin D1, Cancer stemness, ROS, FOXM1

## Abstract

**Background:**

Cancer stem cells (CSCs) require stromal signals for maintaining pluripotency and self-renewal capacities to confer tumor metastasis. Resolvin D1 (RvD1), an endogenous anti-inflammatory lipid mediator, has recently been identified to display anti-cancer effects by acting on stroma cells. Our previous study reveals that hepatic stellate cells (HSCs)-derived cartilage oligomeric matrix protein (COMP) contributes to hepatocellular carcinoma (HCC) progression. However, whether RvD1 inhibits paracrine of cancer-associated fibroblasts (CAFs)-derived COMP to prevent epithelial-mesenchymal transition (EMT) and cancer stemness in HCC remains to be elucidated.

**Methods:**

CAFs were isolated from HCC tissues. Direct and indirect co-culture models were established to analyze the interactions between HCC cells and CAFs in the presence of RvD1 in vitro. The transwell and tumor sphere formation assays were used to determine invasion and stemness of HCC cells. The subcutaneous tumor formation and orthotopic liver tumor models were established by co-implantation of CAFs and HCC cells to evaluate the role of RvD1 in vivo. To characterize the mechanism of RvD1 inhibited paracrine of COMP in CAFs, various signaling molecules were analyzed by ELISA, western blotting, reactive oxygen species (ROS) detection, immunofluorescence staining, dual luciferase reporter assay and chromatin immunoprecipitation assay.

**Results:**

Our data revealed that RvD1 treatment can impede the CAFs-induced cancer stem-like properties and the EMT of HCC cells under co-culture conditions. In vivo studies indicated that RvD1 intervention repressed the promoting effects of CAFs on tumor growth and metastasis of HCC. Furthermore, RvD1 inhibited CAF-induced EMT and stemness features of HCC cells by suppressing the secretion of COMP. Mechanistically, formyl peptide receptor 2 (FPR2) receptor mediated the suppressive effects of RvD1 on COMP and forkhead box M1 (FOXM1) expression in CAFs. Notably, RvD1 impaired CAF-derived COMP in a paracrine manner by targeting FPR2/ROS/FOXM1 signaling to ultimately abrogate FOXM1 recruitment to the COMP promoter.

**Conclusion:**

Our results indicated that RvD1 impaired paracrine of CAFs-derived COMP by targeting FPR2/ROS/FOXM1 signaling to repress EMT and cancer stemness in HCC. Thus, RvD1 may be a potential agent to promote treatment outcomes in HCC.

**Electronic supplementary material:**

The online version of this article (10.1186/s13046-019-1163-6) contains supplementary material, which is available to authorized users.

## Background

Hepatocellular carcinoma (HCC) is one of the fastest-increasing causes of cancer-related deaths in the United States and is one of the most common cancers in China [1, 2]. Although remarkable improvements of therapeutic strategies, such as local ablation, surgery and liver transplantation, have been achieved for early-staged HCC, patients suffering advanced-staged HCC are still deficient in effective strategies to control the malignant progression. One of the main reasons for this bleak prognosis associated with advanced HCC is the high incidence of metastasis [3]. Therefore, it is urgent to clarify novel molecular pathogenesis involved in the invasiveness and metastasis of HCC and to identify new effective therapeutic targets.

Cancer-associated fibroblasts (CAFs) are the most abundant cells in the tumor microenvironment (TME), a key source of the extracellular matrix that contributes to the desmoplastic stroma, and play crucial roles during cancer malignant progression and metastasis [4, 5]. CAFs are found in stroma-rich primary HCC and facilitate proliferation, migration, invasion, epithelial-mesenchymal transition (EMT), therapeutic resistance, and induce cancer stem cell (CSC)-like phenotypes of HCC cells by reshaping the tumor microenvironment and paracrine of a variety of cytokines [6, 7]. For example, peri-tumor tissue-sourced fibroblasts secrete various cytokines, including IL-6, CXCL1, CCL2, SCGF-β, CXCL8 and HGF, to recruit cancer stem cells, maintain cancer stemness and promote intrahepatic metastasis of HCC [8]. Moreover, HCC-derived exosomal miR-1247-3p trans-differentiates lung fibroblasts into CAFs and then educates CAFs to secrete IL-6 and IL-8 to promote cancer stemness, chemoresistance, EMT, and tumorigenicity of HCC cells, leading to lung metastasis of HCC [9]. Nevertheless, the explicit mechanism accounting for the interactions between CAFs and HCC cells is complex and still obscure.

EMT is a key process involved in cancer invasion and distant metastasis, featuring the loss of intercellular adhesion with the downregulation of epithelial markers (such as E-cadherin) and upregulation of mesenchymal markers (such as N-cadherin and vimentin). Cancer stem-like properties are distinguished by the propensity to exhibit high self-renewal, differentiation and tumorigenicity capacities [10]. Although molecular markers of cancer stemness are still emerging, transcription factors including Nanog, Sox2 and Oct4 have been strongly identified as master mediators of pluripotency [11]. In fact, a direct link between the EMT process and the acquisition of stem-like properties by neoplastic cells has been previously reported [12]. Furthermore, cancer stem cells possess a propensity to express lower levels of epithelial marker and higher levels of mesenchymal markers during metastasis [12].

Cartilage oligomeric matrix protein (COMP), a valuable maker of cartilage turnover, is a 524-KDa soluble pentameric glycoprotein [13]. COMP expression in the fibrotic liver is increased by reactive oxygen species (ROS), chemokines, growth factors, matrix stiffness, and matricellular proteins [14, 15]. However, the mechanisms underlying the regulation of COMP expression by ROS and other factors are still not clear. Moreover, COMP enhances the synthesis of type 1 collagen in hepatic stellate cells (HSCs) via CD36 receptor-mediated activation of MEK1/2-pERK1/2 signaling to regulate liver fibrosis [16]. The potential role of COMP in tumors has been reported in breast cancer, prostatic cancer and colon cancer [17–19], and a high expression level of COMP has been detected both in tumor cells and the surrounding stroma. Our previous study has also shown that HSCs-derived COMP facilitates invasion and metastasis of HCC by activating PI3K-AKT and MEK-ERK signaling in a CD36-dependent manner [20]. However, little is known about the regulatory mechanism of COMP expression and its role in maintaining cancer stem-like phenotypes.

Forkhead box M1 (FOXM1), a member of FOX transcription factor family, has a crucial role in cell-cycle progression [21]. Previous studies have shown that FOXM1 is overexpressed in a variety of human malignancies, and most of research has focused on tumor cells including HCC [22]. Recently, studies have unveiled that FOXM1 plays a pivotal role in bleomycin-induced pulmonary fibrogenesis and serves as a driver of lung fibroblast activation [23]. Analogously, deletion of FOXM1 in postnatal cardiomyocytes results in cardiac fibrosis [24], and pulmonary artery smooth muscle cells-specific FOXM1 regulates hypoxia-induced pulmonary hypertension [25]. However, its potential role in the stroma of HCC requires further elucidation. Intriguingly, there seems to be potential links between ROS and FOXM1. FOXM1 can exert its role in a ROS-dependent manner and has been recognized as a critical regulator of oxidative stress during oncogenesis [26].

Resolvins, a family of endogenous proresolving and anti-inflammatory lipid mediators derived from ω-3 polyunsaturated fatty acid, have been generated from EPA and DHA [27, 28]. The potent in vivo actions of RvD1 have been reported in many pathologies, such as obesity and those affecting the vascular, airway, dermal, renal and ocular systems, and in processes including pain, fibrosis and wound healing. Its role in governing neutrophil influx, macrophage resolution and reducing pro-inflammatory mediators seems to be fundamental in all organs [27]. The illuminating view of tumors as “wounds that do not heal” provides insights that RvD1 may exert a critical role in carcinogenesis. Among this family, RvD1 is specially derived from DHA by biosynthetic pathways involving lipoxygenase (LOX) [29], and RvD1 can exert effects through binding to its two receptors, lipoxin A4 receptor/formyl peptide receptor 2 (ALX/FPR2) and a GPCR denoted GPR32 [30]. Resolvins differ from classic anti-inflammatories in that they stimulate, as agonists, the resolution of inflammation, act at significantly lower doses and are not immunosuppressive [27, 31, 32]. Previous studies have confirmed that resolvins facilitate macrophage phagocytosis of debris from apoptotic tumor cells and counterregulate macrophage secretion of pro-inflammatory cytokines in a restricted receptor-dependent manner to suppress debris-stimulated tumor growth, and the antitumor activity of resolvins is mediated by stromal cells rather than a direct action on tumor cells [31]. Interestingly, CAFs, which are a major component of the tumor stroma, play a profound role in forming the inflammatory microenvironment to regulate stroma-tumor interactions in HCC. However, the effects of resolvins on CAFs in HCC are still not clear.

In this study, we postulated that RvD1 could inhibit COMP secretion in CAFs in a paracrine manner via FPR2/ROS/FOXM1 signaling to block stoma-tumor cells interactions and then suppress EMT and cancer stemness to alleviate malignant progression of HCC.

## Methods

### Reagents and antibodies

The COMP ELISA kit, recombinant human COMP protein (rh-COMP) and human COMP neutralization antibody were purchased from R&D systems (Minneapolis, MN USA). RvD1 was obtained from Cayman Chemical Corporation (Ann Arbor, MI, USA). Detailed information about the antibodies utilized in this study is presented in Additional file 1: Table S1. N-acetyl-L-cysteine (NAC) and H_2_O_2_ were purchased from Sigma (St. Louis, MO, USA). Scrambled siRNA (si-Control: sense 5′-UUCUCCGAACGUGUCACGUTT-3′; antisense 5′-ACGUGACACGUUCGGAGAATT-3′) and ALX/FPR2 siRNA (si-FPR2: sense 5′-CGGUUUGUCAUUGGCUUUATT-3′; antisense 5′- UAAAGCCAAUGACAAACCGTT-3′) were purchased form GenePharma (Shanghai, China) as we previously described [33]. Si-Control and si-FOXM1 were obtained from Santa Cruz Biotechnology. The pcDNA3.1-Control and pcDNA3.1-FOXM1 (pcDNA/ FOXM1) were purchased from Invitrogen (USA). Luciferase-expressing lentiviruses were purchased from GeneChem Co, Ltd. (Shanghai, China). All these reagents were stored following the manufacturer’s instructions.

### Cell culture and intervention

Two HCC cell lines (SMMC-7721, Hep3B) were obtained from the Type Culture Collection of the Chinese Academy of Sciences (Shanghai, China), and cultured in DMEM (HyClone, Logan, UT, USA) with 10% fetal bovine serum (FBS, Gibco, USA), 100 μg/mL streptomycin and 100 U/mL penicillin (Sigma, USA) in a humidified atmosphere at 37 °C containing 5% CO2.

### Isolation of CAFs

The methods used to isolate CAFs from HCC tissue have been previously described [34]. Briefly, surgically resected HCC tissues and peritumoral tissues were minced into 2–3-mm fragments, washed in phosphate-buffered saline (PBS), and cultured in 6-well plates supplemented with F12/DMEM containing 10% fetal bovine serum and 1% penicillin/streptomycin. Peritumoral fibroblasts (PTFs), and the CAFs were allowed to grow out of the tissue fragments at 37 °C with 5% CO_2_. After purification, the expression of α-SMA in these cells was determined by immunofluorescence and western blotting. We used CAFs within 6 passages for the various experiments.

### HCC-CAFs co-culture model

Both direct and indirect co-culture models were established to analyze the interactions between hepatocellular carcinoma cells and CAFs in the presence of RvD1. For the indirect HCC-CAFs co-culture model, CAFs were treated with or without RvD1 (400 nM) for 24 h; the cells were then serum-starved (1% serum in fresh medium) for an additional 48 h, and the conditioned medium (CM) of CAFs was subsequently collected and filtered. Then, the CM of CAFs was added to serum-starved HCC cells (SMMC-7721, Hep3B). For the direct HCC-CAF co-culture model, HCC cells and CAFs were proportionally mixed and seeded on the slides in 24-well plates. After the cells were serum-starved for 6–8 h, the cells were treated with RvD1 (400 nM) for 48 h.

### Immunofluorescence staining

After completion of the designated treatment, immunofluorescence staining was performed according to a standard protocol as described in our previous study [35]. The cells on the slides were imaged and recorded with appropriate excitation and emission spectra at a magnification of 400 × using a Zeiss Instruments confocal microscope (Zeiss, Oberkochen, Germany).

### Enzyme-linked immunosorbent assay (ELISA)

CAFs were treated RvD1 at different concentrations (0, 200, 400, and 800 nM) for 24 h; then, the cells were serum-starved (1% serum in fresh medium) for an additional 48 h and the supernatant collected and centrifuged (1500 rpm for 5 min). Subsequently, the secretion of COMP into the conditioned medium was detected using ELISA kits following the manufacturer’s instructions (R&D Systems, USA). The contents of RvD1 in 25 HCC and adjacent nontumor samples were examined using an ELISA kit purchased from Cayman Chemical Corporation (Ann Arbor, MI, USA) as previously described [31].

### Transwell migration and invasion assays

The migration assay was performed using Transwell chambers (BD Biosciences, Franklin Lakes, NJ), in which 2 × 10^4^ cells in 200 μL serum-free medium were plated in the upper chambers. For the invasion assay, the basement membranes of the filters were coated with 50 μL Matrigel (Matrigel; BD Biosciences, Bedford, MA). After completion of the designated intervention, the CAFs were utilized for the migration assay, and HCC cells (SMMC-7721, Hep3B) were used for the invasion assay. Finally, the cells that had migrated or invaded to the lower surface of the membrane were fixed and stained with crystal violet. The results were analyzed by counting the stained cells using optical microscopy (100 × magnification) in five randomly selected fields. Each experiment was carried out in triplicate wells and repeated at least three times.

### Tumor sphere formation assay

After co-culturing with the CM collected from CAFs, HCC cells were seeded at a density of 5000 cells per well in six-well ultralow attachment plates (Corning, Corning, NY, USA) and then incubated with serum-free DMEM/F12 medium (Gibco) supplemented with 1% B27 (Invitrogen, Carlsbad, CA, USA), 20 ng/mL human FGF and 20 ng/mL human EGF. The cells were subsequently cultured at 37 °C with 5% CO_2_ for two weeks. A microscope (Nikon Instruments Inc.) was utilized to count the number and measure the diameter of the tumor sphere at a magnification of 200 × .

### Intracellular ROS measurement

Intracellular ROS production in CAFs was determined using the ROS probe 2,7-dichlorofluoresceindiacetate (DCF-DA). Briefly, after treatment with RvD1, NAC or H_2_O_2_, CAFs were incubated with F12/DMEM containing DCF-DA (10 μmol/L) for 30 min at 37 °C, and the DCF-DA fluorescence intensity was measured by flow cytometry using a FACSCalibur (BD Biosciences, San Diego, CA, USA), or using a Zeiss Instruments confocal microscope to visualize the ROS level in CAFs.

### Cell viability assay

SMMC-7721, Hep3B were seeded into 96-well plates at a density of 5 × 10^3^ cells per well and treated with conditioned medium (CM_CAFs,_ CM_CAFs + RvD1_), CM_CAFs_ + anti-COMP and CM_CAFs + RvD1_ + rh-COMP for 24, 48, 72 and 96 h. Subsequently, the MTT assay was applied to assess the cell viability, and the absorbance at 490 nm detected by using a multiwall microplate reader (BIO-TEC Inc., VA) was used for the assessment.

### Colony formation assay

After finishing the designated treatment, the cells were plated in a six-well plate (1000 cells per well) and cultured in fresh medium for 14 days. The plates were washed with phosphate-buffered saline (PBS), fixed in 4% formalin, stained with crystal violet solution for 15 min and then washed with PBS to remove excess dye. The number of colonies was counted for each sample.

### Quantitative real-time PCR (qRT-PCR)

Total cellular RNA was extracted using TRIzol reagents (Takara Bio, Dalian, China) and quantitated by the absorbance at 260 nm. The RNA (1 μg) sample was reverse-transcribed with PrimeScript RT Master Mix, and quantitative real-time PCR was conducted with SYBR-Green PCR Master Mix (Takara Bio, Dalian, China) using gene-specific primers. The sequences of the specific primers are presented in Additional file 2: Table S2. GAPDH was used as a loading control, and the results were calculated using the 2^-ΔΔCt^ method.

### Western blot analysis

Total proteins of CAFs, SMMC-7721 and Hep3B (1 × 10^6^) grown under our experimental conditions were extracted using RIPA Lysis Buffer (Beyotime, Guangzhou, China). The BCA protein assay kit (Pierce, Rockford, USA) was utilized to measure the concentration of the proteins based on the manufacturer’s instructions. The details of the western blot assay have been previously described [36]. The immunoreactive bands to visualize the expression of designated proteins were evaluated using the chemiluminescence detection system through the peroxidase reaction, and the images of the bands were recorded with the ChemiDoc XRS imaging system (Bio-Rad, USA). β-actin was used as the internal loading control.

### Transfection

Plasmids and siRNAs were transiently transfected into CAFs cultured in a 6-well plate at a density of 2 × 10^5^ per well using lipofectamine 2000 (Invitrogen, CA, USA). For transient transfection, CAFs were transfected with plasmids or siRNAs at different concentrations according to the manufacturers’ instructions for 48 h prior to further experiments. The luciferase-expressing lentiviruses were transfected into Hep3B cells following the standard protocols from GeneChem Co, Ltd.

### Construction of COMP promoter reporter plasmids and mutagenesis

The final full-length reporter plasmid containing multiple FOXM1-binding sites was designated pLuc–COMP-#1 and purchased from Genecopoeia (#HPRM30080 Guangzhou, China). The deletion mutation reporter plasmid, which did not have FOXM1-binding sites, designated as pLuc–COMP-#2, was then generated. Both constructs were verified by sequencing the inserts and flanking regions of the plasmids.

### Dual luciferase reporter assay

CAFs were transfected with the indicated COMP promoter reporter (pLuc–COMP-#1 and pLuc–COMP-#2 respectively), siFOXM1, or overexpression-FOXM1 plasmid in different groups. The COMP promoter activity was normalized via co-transfection of a β-actin/Renilla luciferase reporter containing the full-length Renilla luciferase gene. The luciferase activity in the CAFs was quantified using a dual luciferase assay system (Promega) for 24 h after transfection.

### Chromatin immunoprecipitation (ChIP)

The ChIP assay were performed as previously described [37]. Briefly, the ChIP assay was conducted using a commercial kit (Upstate Biotechnology) based on the manufacturer’s instructions. The PCR primers are indicated in Additional file 2: Table S2.

### In vivo tumorigenesis assays

All animal experiments were performed according to the protocols sanctified by the ethical committee of Xi’an Jiaotong University. For the subcutaneous tumor formation assay, 5 × 10^5^ of Hep3B cells were suspended in 100 μL PBS alone in the control group, and 100 μL of Hep3B cells (5 × 10^5^) and CAFs (5 × 10^5^), mixed in a single cell suspension, was subcutaneously injected into the left flanks of 4-week-old male BALB/c nude mice (obtained by and housed in the Animal Center at Medical College, Xi’an Jiaotong University). Then, the animals that received the Hep3B and CAF co-injection were randomly divided into two groups (six mice per group). One group received RvD1 (6 μg/kg/d for 4 weeks), and the other group was treated with vehicle control. Tumor growth was continuously monitored by calculating the tumor volume according to the following formula: V (tumor volume) =0.5 × s (shorter diameter)^2^ × L (longer diameter). The mice were sacrificed at day 28, and the tumor samples were weighed, measured and then stained by immunohistochemistry for histological analyses. The immunohistochemistry procedure was performed as we previously reported [38].

An orthotopic liver tumor model in nude mice was established to assess metastasis according to our previous report [39]. Briefly, Hep3B cells were transfected with luciferin lentiviruses, and subsequently, 5 × 10^5^ transfected-Hep3B cells mixed with 5 × 10^5^ CAFs and suspended in 100 μL PBS were injected into nude mouse liver. The mice (six mice per group) were then treated with RvD1 (6 μg/kg/d for 3 weeks) or vehicle through intraperitoneal injection. After 3 weeks, bioluminescence imaging (BLI) was conducted to monitor the tumor volume and metastases after injection of 450 mg/kg D-luciferin substrate (Biosynth, Naperville, IL, USA) in PBS into anesthetized mice.

### Statistical analysis

All the data are displayed as the mean ± standard deviation (SD) of three independent experiments. The Student’s t-test was applied to compare two groups. Statistical analyses for multiple comparisons were conducted using one-way ANOVA followed by the LSD post hoc test with SPSS 18.0. *, *P* < 0.05 and **, *P* < 0.01 were considered to indicate a statistically significant difference.

## Results

### RvD1 pretreatment impedes CAFs-induced cancer stem-like properties and metastasis of HCC

**Fig. 1:**
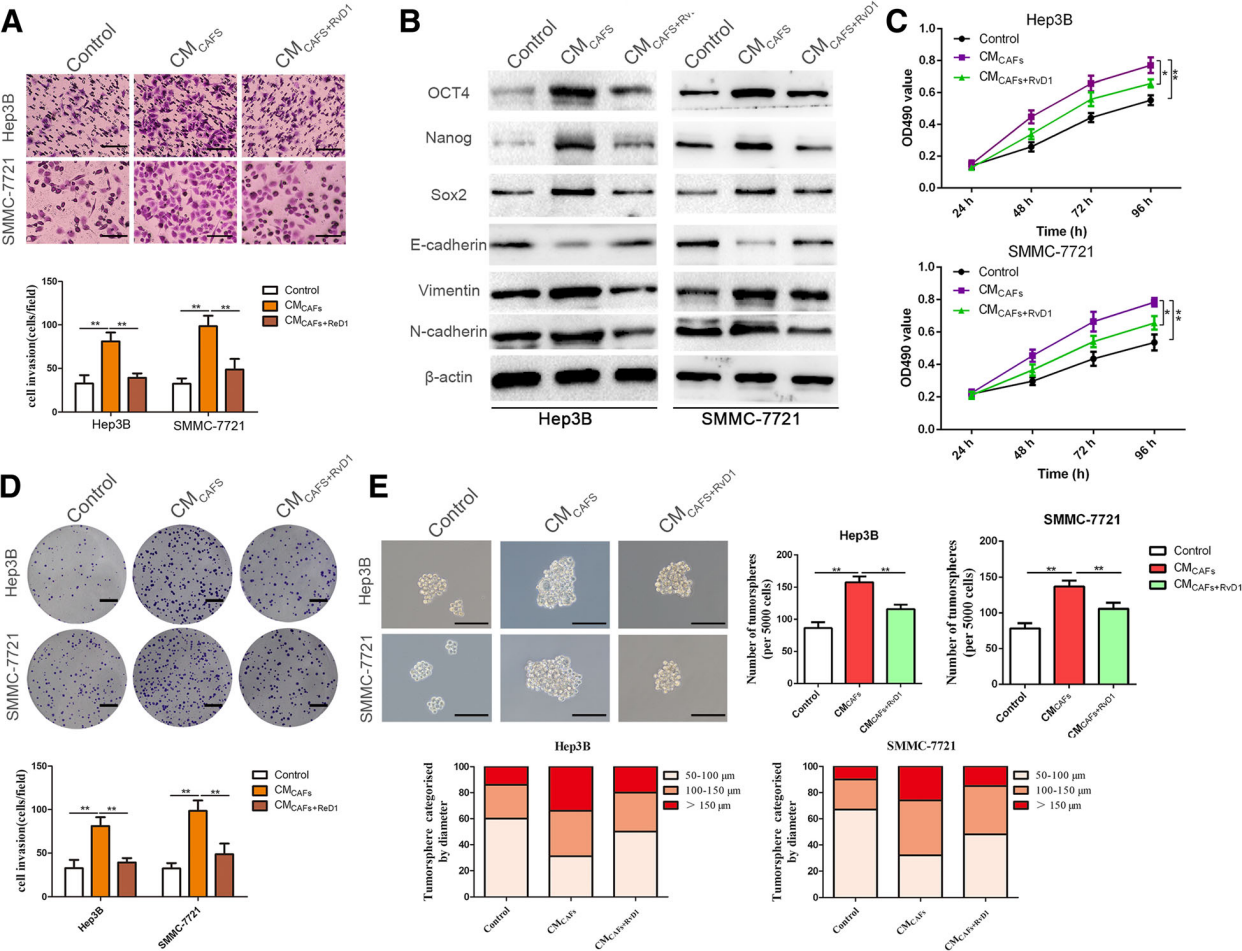
RvD1 impedes the CAFs-induced cancer stemness and EMT of HCC cells. (**a**) Hep3B and SMMC-7721 cells were incubated with CM from CAFs (CM_CAFs_) or CM from CAFs pre-treated with RvD1(400 nM) (CM_CAFs + RvD1_) for 24 h, The cells were then seeded in a matrigel-coated invasion chamber for 24 h. The invasive cells were quantified by counting the number of cells in 5 random fields at × 100 magnification. Scale bars = 50 μm. n = three independent experiments, **P < 0.01 by ANOVA. (**b**) Hep3B and SMMC-7721 cells were treated with CM_CAFs_ and CM_CAFs + RvD1_ for 48 h, and western blotting analysis was performed to test the expression of stemness markers (OCT4, Nanog, Sox2), and epithelial-mesenchymal transition markers (E-cadherin, N-cadherin, vimentin). (**c**) Hep3B and SMMC-7721 cells were incubated with CM_CAFs_ and CM_CAFs + RvD1_ for 24, 48, 72 and 96 h, and cell viability was evaluated by MTT assay. n = three independent experiments, *P < 0.05 or **P < 0.01 by ANOVA. (**d**) Effects of CM_CAFs_ and CM_CAFs + RvD1_ on the colony-forming ability of Hep3B and SMMC-7721 cells were assessed by colony formation assay. Images are representative of three independent experiments, and the colony number was counted and plotted. Scale bar = 1 cm. n = three independent experiments, **P < 0.01 by ANOVA. (E) Tumorsphere formation assay of Hep3B and SMMC-7721 cells inculated with CM_CAFs_ or CM_CAFs + RvD1_. The number and size of tumorspheres were counted, measured and plotted. Magnification is × 200, and scale bars = 50 μm. n = three independent experiments, **P < 0.01 by ANOVA

The identification of CAFs was confirmed by immunofluorescence staining, qRT-PCR and western blotting. As shown in Additional file 3: Figure S1, higher expression levels of α-SMA, collagen 1α and fibronectin were observed in CAFs than in PTFs (peritumoral fibroblasts), consistent with previous studies [34]. To evaluate whether CAFs impacted the malignant phenotypes of HCC cells, Hep3B and SMMC-7721 cells were subjected to further experiments. CAFs conditioned medium (CM_CAFs_) treatments for 24 h induced EMT and enhanced the invasive capacities of Hep3B and SMMC-7721 cells (*P* < 0.05, Fig. 1a, b and Additional file 4: Figure S2A). Similarly, the colony formation and MTT assays further revealed that Hep3B and SMMC-7721 cells co-cultured with CM_CAFs_ showed higher proliferation ability compared with control cells (P < 0.05, Fig. 1c and d). Next, we used an ELISA kit to quantify the content of RvD1 in HCC, and we found that the content of RvD1 in HCC tissues was significantly decreased compared with the adjacent nontumor samples (from 25 paired HCC and adjacent nontumor samples) (*P* < 0.05, Additional file 5: Figure S3A). In humans, many of the prominent DHA-derived resolvins are primarily synthesized through the interaction of 15-LOX with 5-LOX (Additional file 5: Figure S3B). Interestingly, as the key enzyme for synthetic RvD1, the expression of 15-LOX was down-regulated in HCC tissues (*P* < 0.05, Additional file 5: Figure S3C). To explore the effects of RvD1 on tumor cells, we treated Hep3B and SMMC-7721 cells with exogenous RvD1 and found that the antitumor activity of RvD1 was unlikely to be mediated by a direct antiproliferative or anti-invasive activity on tumor cells (Additional file 6: Figure S4), which was in accordance with previous discoveries [31]. Then, CAFs were pretreated with RvD1 (400 nM), and conditioned medium (CM_CAFs + RvD1_) was collected to treat Hep3B and SMMC-7721 cells. Our data indicated that RvD1 treatment repressed the promotion-effects of CAFs on HCC cell invasion and proliferation (*P* < 0.05, Fig. 1a-d). The tumor sphere formation assay was utilized to assess the efficacy of RvD1 to affect CAFs-elicited stem-like characteristics of HCC cells. A significant increase in mammosphere formation efficiency was observed in SMMC-7721 and Hep3B cells exposed to CM_CAFs_ treatment compared with the control group (*P* < 0.05, Fig. 1e). In contrast, pretreatment of CAFs with RvD1(400 nM) effectively reversed this promoting effect on mammosphere formation (*P* < 0.05, Fig. 1e). In addition, CM_CAFs_ treatment induced CSC marker expression in Hep3B and SMMC-7721 cells, as revealed by increases in Nanog, Sox2, Oct4, CD44, EpCAM and CD90 expression, whereas these effects of CM_CAFs_ were also partly hindered by CAFs pretreatment with RvD1(400 nM) (*P* < 0.05, Fig. 1b and Additional file 4: Figure S2).

**Fig. 2:**
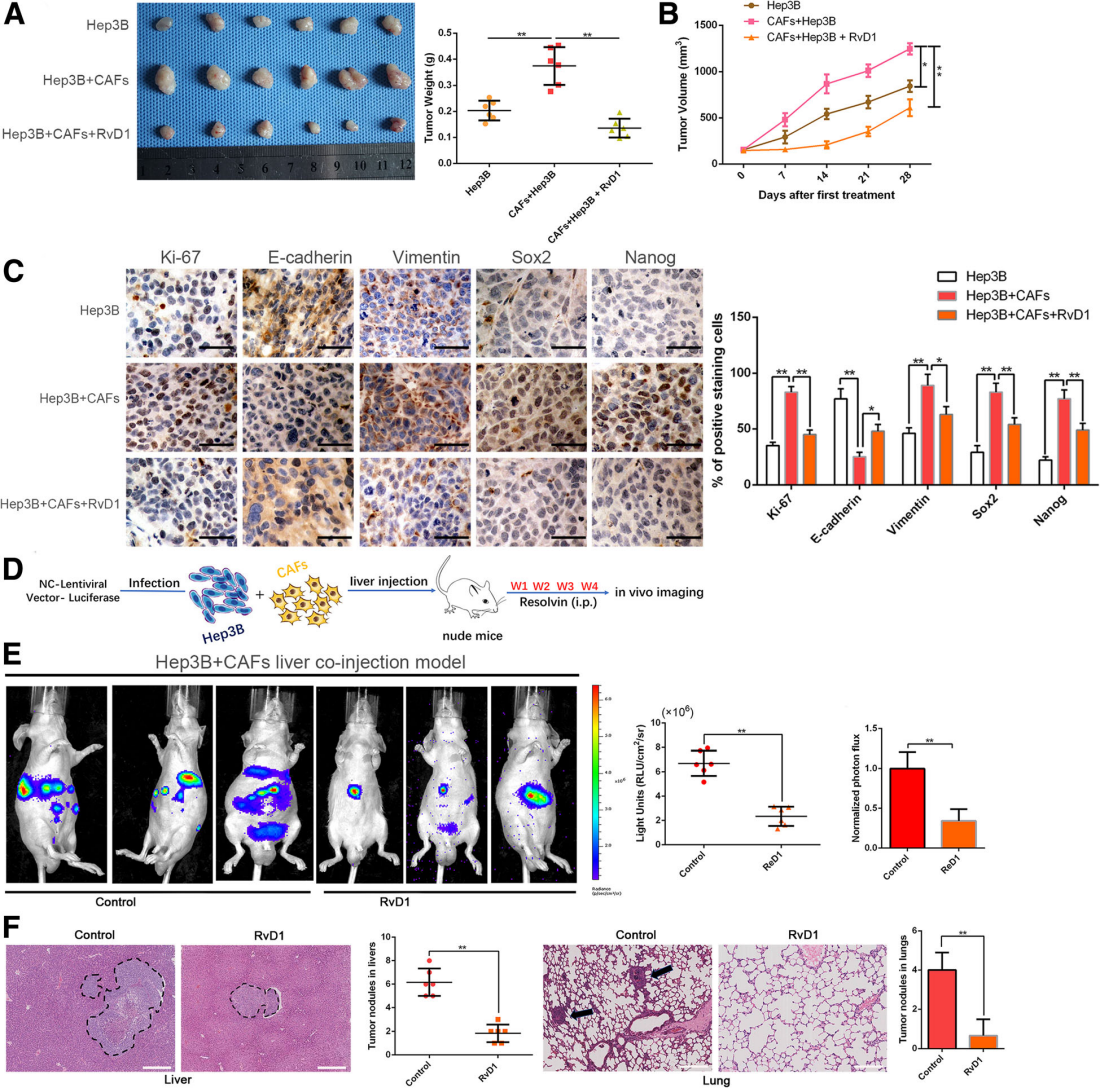
RvD1 inhibits the CAFs-induced tumorgenicity of Hep3B cells along with reversing the mesenchymal and stem-like phenotypes. (**a**) Representative images of subcutaneous xenografts in nude mice implanted with Hep3B in the presence or absence of CAFs and treated with RvD1 (*n* = 6). (**b**) Xenografts weight (mg) and tumor sizes were monitored and undergone quantification analysis. n = 6, **P < 0.01 by ANOVA for tumor weight; *P < 0.05 or **P < 0.01 by repeated-measures ANOVA for tumor sizes. (**c**) Immunohistochemistry staining and the semi-quantification data of Ki-67, E-cadherin, Vimentin, Sox2 and Nanog in xenograft tissues from different groups. Magnification is × 400, the scale bar represents 20 μm. n = 6, *P < 0.05 or **P < 0.01 by ANOVA. (**d**) Schematic of establishing the orthotopic liver tumor model in nude mice using Luciferase-expressing Hep3B cells admixed with CAFs co-injection and administrated with RvD1 (n = 6 per group). (**e**) Representative BLI images of orthotopic liver tumor and metastasis in vivo were shown, and Luciferase signaling was analyzed. n = 6, **P < 0.01 by t test. (**f**) Representative hematoxylin and eosin–stained sections, and quantification analysis of tumor nodules in livers and metastasis nodules in lungs were shown. The scale bar represents 365 μm. n = 6, **P < 0.01 by t test

CSCs feature stem cell-like properties, including self-renewal, tumor initiation, proliferation, invasion and metastasis [40]. Therefore, we investigated whether CAFs could promote in vivo tumorigenesis by subcutaneously injecting Hep3B cells with or without CAFs according to a 1:1 ration in nude mice. Co-implantation with Hep3B cells and CAFs produced a higher tumor weight and volume compared with the control group (*P* < 0.05, Fig. 2a and b). Analogously, administration of RvD1 (6 μg/kg/d) into mice co-implanted with Hep3B cells and CAFs clearly inhibited the promoting effect of CAFs on tumorigenesis (P < 0.05, Fig. 2a and b). Moreover, immunohistochemistry staining and semiquantification of the IHC data showed that RvD1 treatment reduced the expression of EMT and CSC makers in subcutaneous tumors arising from mice co-implanted with Hep3B cells and CAFs (Fig. 2c). Additionally, we further established the orthotopic liver tumor model in nude mice using Hep3B cells admixed with CAFs to explore the role of RvD1 in the spontaneous metastatic growth of HCC in vivo (Fig. 2d). As shown in Fig. 2e-f, both the bioluminescence intensity and metastatic nodules indicated that CAFs significantly promoted tumor growth and metastasis of HCC in vivo, while RvD1 suppressed this process (*P* < 0.05).

### RvD1 represses the secretion of CAFs-derived COMP and downregulates ROS levels and FOXM1 expression

**Fig. 3:**
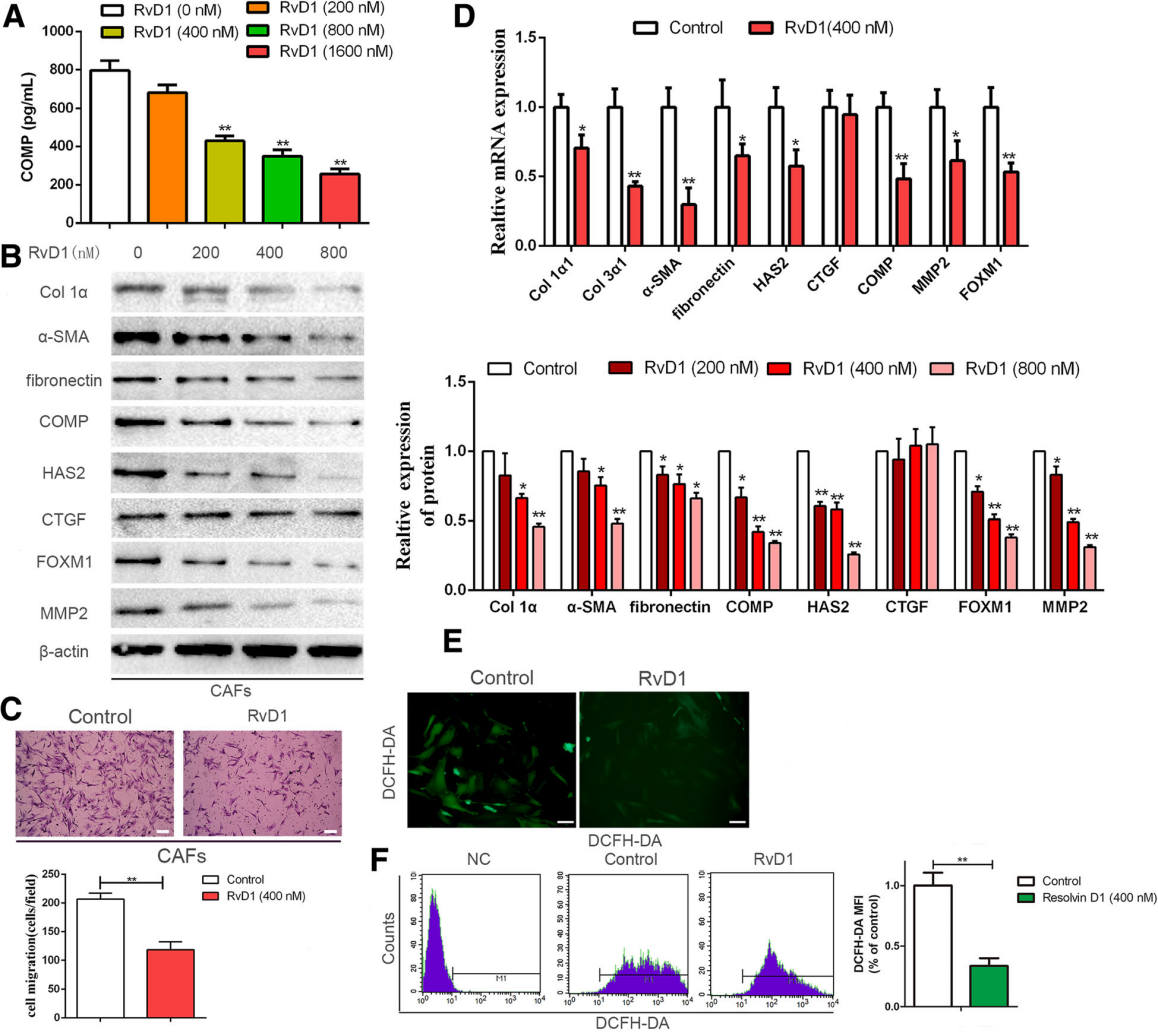
RvD1 abolishes secretion of COMP, down-regulates ROS level and suppresses FOXM1 expression in CAFs. (**a**) CAFs were treated with RvD1 (0, 200, 400 and 800 nM) for 24 h, then serum-starved for an additional 48 h, and the CAFs culture media was used to detect the secretion of COMP by Elisa assay. n = three independent experiments, **P < 0.01 by ANOVA. (**b**) CAFs were administrated with RvD1 (0, 200,400 and 800 nM) for 48 h, western blotting was used to detect the expression of Col 1α1, Col 3α1, α-SMA, fibronectin, HAS2, CTGF, COMP, MMP2 and FOXM1. *n* = three independent experiments, **P < 0.01 by ANOVA. (**c**) The migration capacity of CAFs in response to RvD1 (400 nM) was detected by Transwell-migration assay. Magnification is × 100, and scale bars = 50 μm. n = three independent experiments, **P < 0.01 by t test. (**d**) CAFs were administrated with RvD1(0, 400 nM) for 24 h, the expression of Col 1α1, Col 3α1, α-SMA, fibronectin, HAS2, CTGF, COMP, MMP2 and FOXM1 at mRNA level were assessed by qRT-PCR. n = three independent experiments, **P < 0.01 by t test. (**e** and **f**) CAFs were treated with RvD1, and the intracellular ROS level was determined by immunofluorescence analysis and flow cytometry using DCF-DA probe. Magnification is × 400, and scale bars = 20 μm. n = three independent experiments, **P < 0.01 versus control by t test

A previous study has illustrated that the antitumor activity of resolvins is mediated by stromal cells instead of by a direct action on tumor cells [23]. Additionally, we have recently demonstrated that HSCs-derived COMP facilitates invasion and metastasis of HCC [20]. Therefore, the regulatory effect of RvD1 on CAFs-derived COMP was evaluated. We treated CAFs with a series of gradually increasing concentrations of RvD1 (0, 200, 400 and 800 nM) for 24 h, cultured the cells in serum-starved medium for 48 h and collected the CM to evaluate the secretion of COMP by ELISA. Our results suggested that RvD1 decreased the secretion of human COMP in a dose-dependent manner (*P* < 0.05, Fig. 3a), consistent with results confirmed by western blotting (*P* < 0.05, Fig. 3b). Moreover, RvD1 not only repressed COMP expression, but it inhibited the expression of α-SMA, collagen 1α, fibronectin, MMP2 and FOXM1 in a concentration-dependent manner (*P* < 0.05, Fig. 3b). According to these results, we selected 400 nM as the designated concentration of RvD1 for further experiments. The treatment with 400 nM RvD1 clearly inhibited the migratory ability of CAFs (*P* < 0.05, Fig. 3c). The qRT-PCR results showed that treatment with 400 nM RvD1 for 24 h effectively reduced the transcriptional levels of collagen 1α1, collagen 3α1, α-SMA, fibronectin, HAS2 (hyaluronan synthase 2), COMP, MMP2 and FOXM1 in CAFs (*P* < 0.05, Fig. 3d). Since RvD1 inhibited FOXM1 expression, we further examined the regulatory effects of RvD1 on its nuclear localization by immunofluorescence analysis. We found that RvD1 suppressed the nuclear localization of FOXM1 in CAFs (Additional file 7: Figure S5A). Double immunofluorescence staining also showed that RvD1 repressed the expression of α-SMA and COMP in CAFs (Additional file 7: Figure S5B). In addition, studies have validated the increased expression of COMP in fibrotic liver in response to ROS [14, 15]. Hence, we assessed ROS production in response to RvD1 treatment. As shown in Fig. 3e and f, ROS levels were significantly decreased under RvD1 treatment compared with the control group in CAFs (*P* < 0.05).

### RvD1 inhibits CAF-induced stem cell-like phenotypes in HCC cells via targeting COMP

**Fig. 4:**
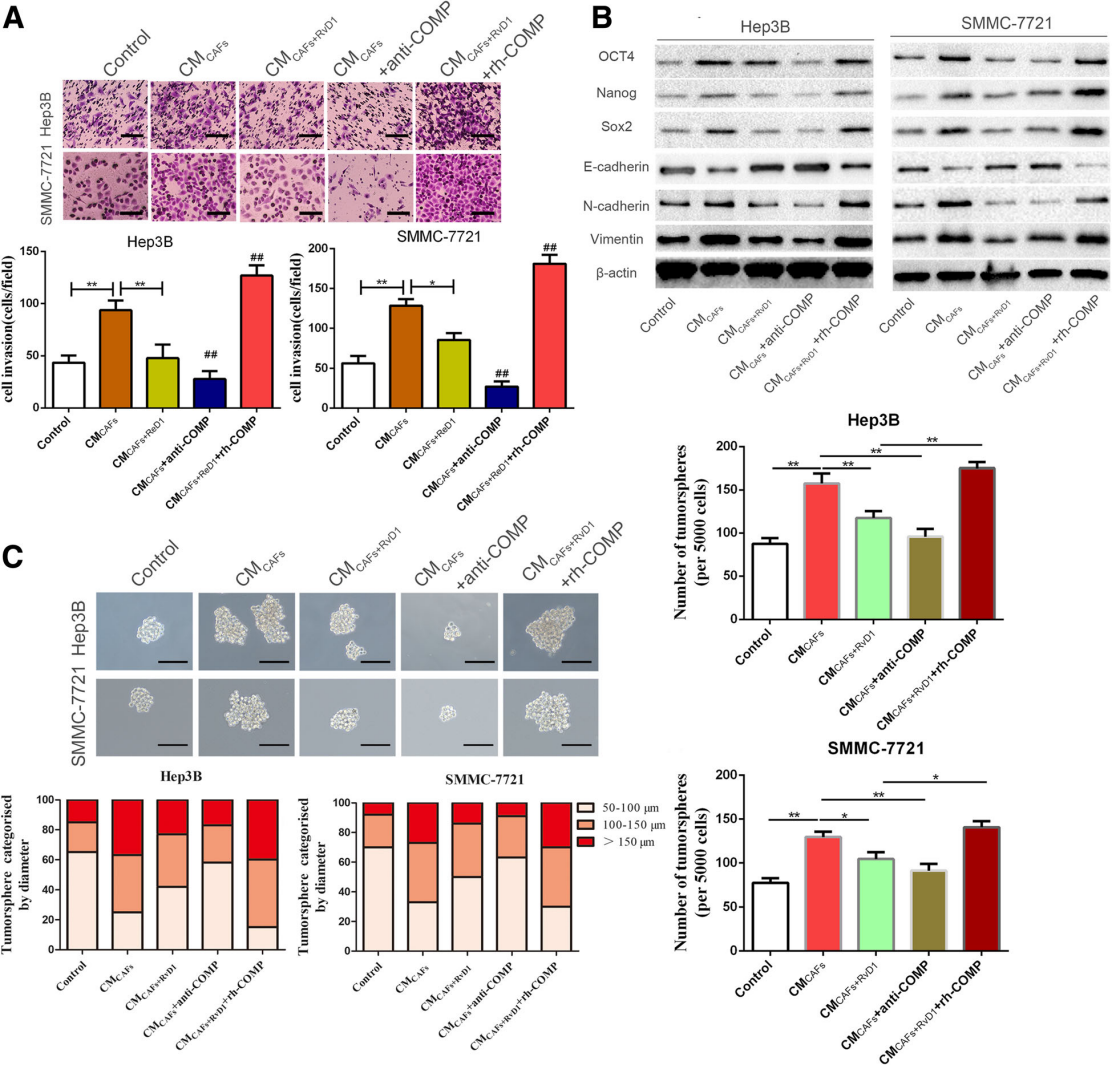
RvD1 suppresses the CAFs-derived COMP mediated cancer stemness of HCC cells. (**a**) Hep3B and SMMC-7721 cells were incubated with CM_CAFs_, CM_CAFs + RvD1_, CM_CAFs_ + anti-COMP and CM_CAFs + RvD1_ + rh-COMP for 24 h, then the invasive ability of HCC cells were evaluated by the Matrigel-invasion assay. Magnification is × 100, and scale bars = 50 μm. n = three independent experiments, **P < 0.01 by ANOVA. (**b**) The expression of CSC and EMT markers after CM_CAFs_, CM_CAFs + RvD1_, CM_CAFs_ + anti-COMP and CM_CAFs + RvD1_ + rh-COMP treatments were determined by western blotting. (**c**) Representative images of the tumorsphere formation assay after CM_CAFs_, CM_CAFs + RvD1_, CM_CAFs_ + anti-COMP and CM_CAFs + RvD1_ + rh-COMP treatments in Hep3B and SMMC-7721 cells. Magnification is × 200, and scale bars = 50 μm. The number of tumorspheres was counted and plotted, and the percentage of tumorspheres with diameters of 50–100 μm, 100–150 μm or > 150 μm was calculated and plotted. Magnification, × 200.The scale bar represents 50 μm. n = three independent experiments, * P < 0.05 or ** P < 0.01versus control by ANOVA

F-actin, a marker of the cell skeleton, shows morphological changes in CAFs and plays a crucial role in cell motility during EMT in tumor cells. Therefore, we examined F-actin expression by immunofluorescence staining during direct HCC-CAF co-culture on slides with or without RvD1 treatment. Our results showed that RvD1 inhibited F-actin expression in the direct HCC-CAFs co-culture model (Additional file 8: Figure S6). COMP is a type of secreted protein that exerts its function through binding to the COMP receptor to influence the cellular phenotypes [20]. To explore whether paracrine COMP signaling played a role in the mechanism by which RvD1 repressed the stem cell-like phenotypes in HCC cells, COMP neutralizing antibody and exogenous rh-COMP were utilized to modulate the concentration of COMP in the CM of CAFs. CM_CAFs_ supplemented with an anti-COMP neutralizing antibody (4 μg/mL) was used to treat Hep3B and SMMC-7721 cells for 24 h. Anti-COMP neutralizing antibody attenuated CM_CAFs_-induced EMT progression and invasive capacity (*P* < 0.05, Fig. 4a, b and Additional file 9: Figure S7A). Similarly, the MTT assay further showed that Hep3B and SMMC-7721 cells co-cultured with CM_CAFs_ containing anti-COMP neutralizing antibody showed a reduced proliferation ability compared with the control groups (*P* < 0.05, Additional file 9: Figure S7B). Moreover, the results of the tumor sphere formation assay showed that both the size and number of tumor spheres in Hep3B and SMMC-7721 cells were surprisingly decreased after co-culture with CM_CAFs_ containing anti-COMP neutralizing antibody (*P* < 0.05, Fig. 4c). Interestingly, when rh-COMP (10 μg/mL) was administered after CM_CAFs + RvD1_ treatment, the expression levels of EMT-associated markers, invasive capacity, cell viability, tumor sphere formation ability and CSC-associated markers were reversed (*P* < 0.05, Fig. 4a-c and Additional file 9: Figure S7). These data convincingly illustrated that RvD1 attenuated CAFs-mediated cancer stem-like phenotypes through the inhibition of paracrine COMP signaling.

### RvD1 suppresses the expression of COMP and FOXM1 and ROS levels in a receptor-dependent manner

**Fig. 5:**
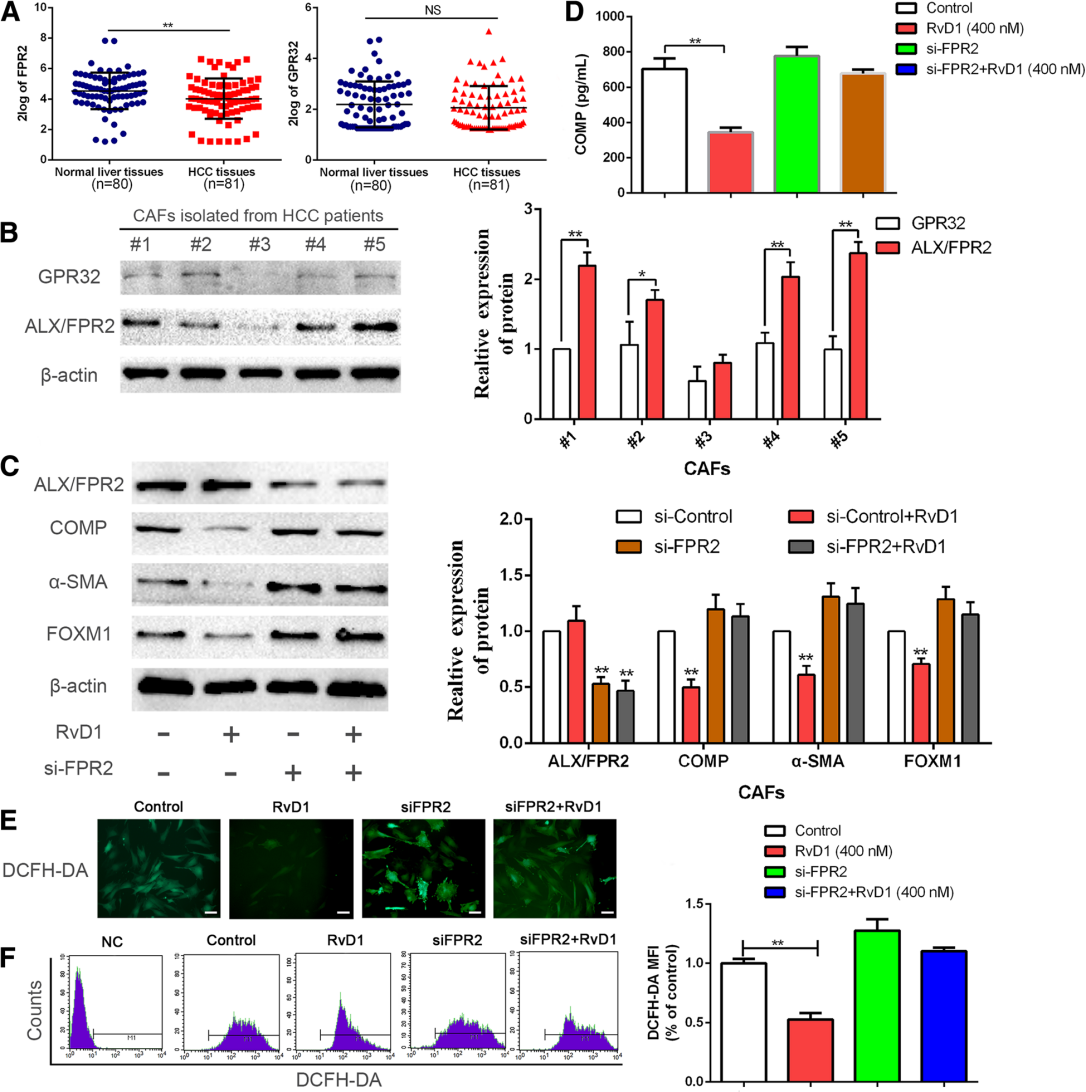
RvD1 suppressed the expression of COMP, FOXM1 and ROS level mediated by ALX/FPR2. (**a**) The expression of ALX/FPR2, one of RvD1receptor, in HCC tissues was significantly lower than that in normal liver tissues, while the expression of GPR32 was no significant difference. Data from a database named R2: Genomics Analysis and Visualization Platform (http://r2.amc.nl). **P < 0.01by t test. (**b**) The expression of ALX/FPR2 and GPR32 in CAFs isolated from five HCC patients were determined by western blotting. * *P* < 0.05 or ** *P* < 0.01 by t test. (**c**) CAFs were transfected with siRNA targeting ALX/FPR2 (si-FPR2) or negative control (si-NC), and 24 h later, 400 nM RvD1 or vehicle were utilized to treat these cells for 48 h. Then ALX/FPR2, α-SMA, COMP and FOXM1 were detected by western blotting. *n* = three independent experiments, ** *P* < 0.01 by ANOVA. (**d**) CAFs were transfected with siRNA targeting ALX/FPR2 (si-FPR2) or negative control (si-NC), and 24 h later, 400 nM RvD1 or vehicle were utilized to treat these cells for 24 h, then serum-starved for an additional 48 h, and the CAFs culture media was used to detect the secretion of COMP by Elisa assay. *n* = three independent experiments, ** *P* < 0.01 by ANOVA. (**e**-**f**) CAFs were transfected with si-FPR2 then treated with RvD1, and the intracellular ROS level was determined by immunofluorescence analysis and flow cytometry analysis using DCF-DA probe. Magnification is × 400, and scale bars = 20 μm. *n* = three independent experiments, ** *P* < 0.01 by ANOVA

Next, we aspired to investigate the further mechanisms of RvD1 regulating the expression of COMP in CAFs. Previous studies have shown that RvD1 exerts effects through binding to its receptors: lipoxin A4 receptor/formyl peptide receptor 2 (ALX/FPR2) and a GPCR denoted GPR32 [30]. First, we detected the expression of ALX/FPR2 and GPR32 in a database named R2: Genomics Analysis and Visualization Platform (http://r2.amc.nl). The results showed that the expression of ALX/FPR2 was significantly downregulated in HCC compared with normal liver tissues (*P* < 0.05, Fig. 5a). However, there was no significant difference in GPR32 expression between HCC and normal liver tissues (Fig. 5a). Furthermore, we examined the expression of ALX/FPR2 and GPR32 in CAFs isolated from five patients by western blotting. Our data showed that GPR32 expression was significantly reduced compared with ALX/FPR2 expression (*P* < 0.05, Fig. 5b). Hence, we postulated that RvD1 suppressed the expression of COMP, ROS and FOXM1, possibly through ALX/FPR2. We verified this hypothesis by depleting ALX/FPR2. The knockdown efficiency of ALX/FPR2 by si-FPR2 was confirmed by western blotting and immunofluorescence staining (*P* < 0.05, Fig. 5c and Additional file 10: Figure S8A). In consistent with our hypothesis, FPR2 depletion partly hindered the efficiency of RvD1 in the repression of COMP, α-SMA, and FOXM1 in CAFs (*P* < 0.05, Fig. 5c, d and Additional file 10: Figure S8A). We further examined the effects of FPR2 depletion on RvD1-induced inhibition of FOXM1 nuclear localization. Immunofluorescence analysis revealed that FPR2 depletion reversed RvD1 mediated-suppression of FOXM1 nuclear localization (Additional file 10: Figure S8B). In addition, FPR2 knockdown impeded RvD1-induced inhibition of ROS levels in CAFs (*P* < 0.05, Fig. 5e and f). Collectively, these data demonstrated that RvD1-mediated inhibition of COMP, FOXM1 and ROS levels occurred in an ALX/FPR2-dependent manner.

### Manipulation of ROS levels regulates the inhibitory efficacy of RvD1 toward COMP and FOXM1

**Fig. 6:**
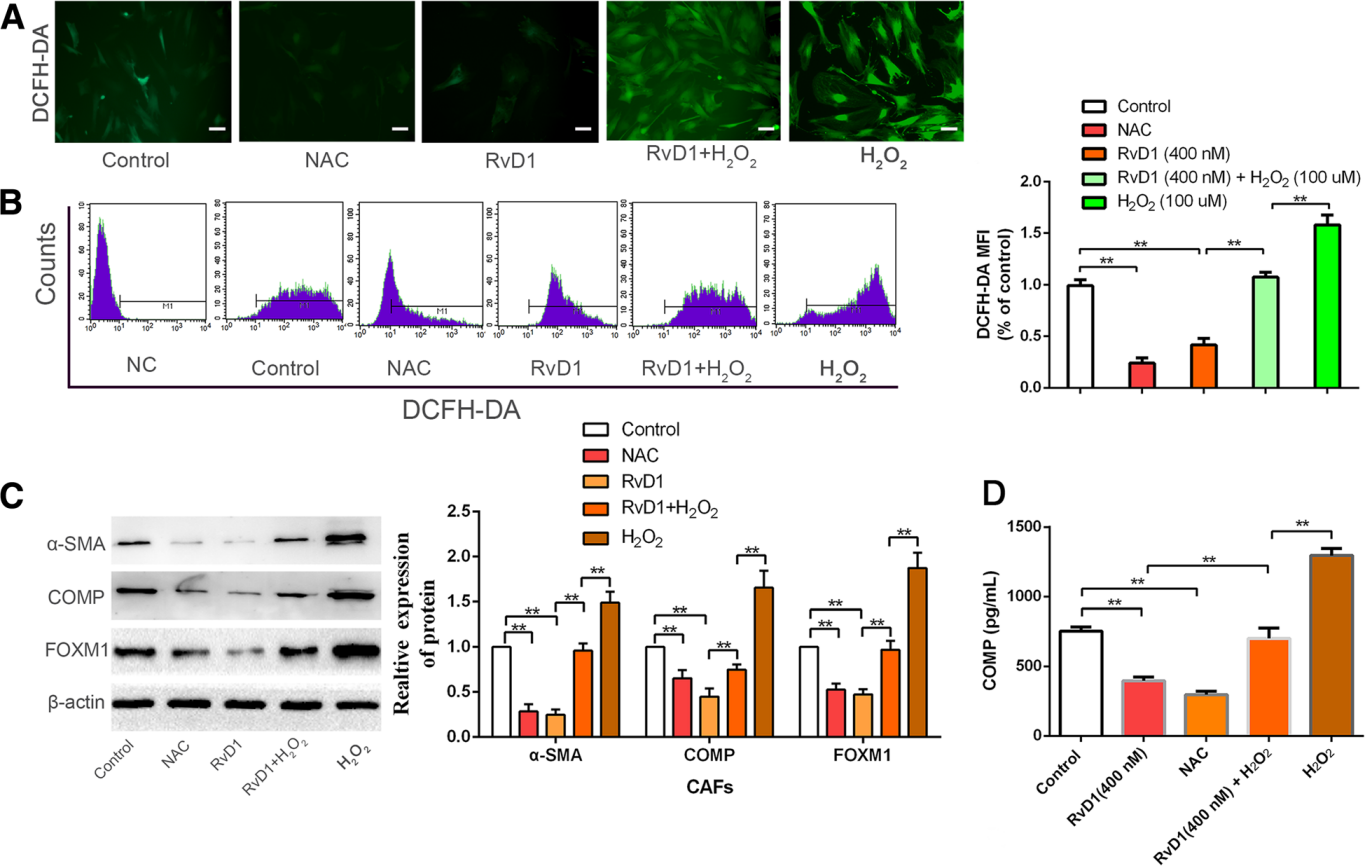
Manipulation of ROS level influenced the efficacy of RvD1 on the expression of COMP and FOXM1. (**a** and **b**) CAFs were treated with RvD1, NAC, RvD1 + H_2_O_2_, and H_2_O_2_, and the intracellular ROS level was determined by immunofluorescence analysis and flow cytometry analysis using DCF-DA probe. Magnification is × 400, and scale bars = 20 μm. n = three independent experiments, ** P < 0.01 by ANOVA. (**c**) CAFs were treated with RvD1, NAC, RvD1 + H_2_O_2_, and H_2_O_2_ for 48 h, the expression of α-SMA, COMP and FOXM1 were detected by western blotting. n = three independent experiments, ** P < 0.01 by ANOVA. (**d**) CAFs were treated with RvD1, NAC, RvD1 + H_2_O_2_, and H_2_O_2_, and the serection of COMP was detected by Elisa assay. n = three independent experiments, ** P < 0.01 by ANOVA

COMP expression in fibrotic liver is increased by ROS, and FOXM1 has been demonstrated to be a critical regulator of oxidative stress during oncogenesis. Our results also demonstrated that RvD1 reduced ROS levels. Thus, to further elucidate the molecular mechanisms involved in RvD1-induced inhibition of FOXM1 and COMP in CAFs, an ROS scavenger, NAC (10 mM), and exogenous reactive oxygen species, H_2_O_2_ (50 μM), were used to manipulate the ROS level. We found that treatment with NAC and RvD1 abolished ROS generation (*P* < 0.05, Fig. 6a and b) and resulted in a remarkable decrease in COMP, α-SMA, collagen 1α, fibronectin, and FOXM1 expression (P < 0.05, Fig. 6c, d and Additional file 11: Figure S9A), inducing an obvious inhibition of FOXM1 nuclear localization (Additional file 11: Figure S9B) in CAFs. Conversely, upregulation of ROS levels induced by H_2_O_2_ led to robust nuclear localization of FOXM1 (Additional file 11: Figure S9B) and facilitated the expression of COMP, α-SMA, collagen 1α, and fibronectin (*P* < 0.05, Fig. 6c, d and Additional file 11: Figure S9A). These results supported our hypothesis that manipulation of ROS levels could regulate the inhibitory efficacy of RvD1 toward COMP and FOXM1 expression in CAFs.

### Abrogation of FOXM1 recruitment is important for RvD1-mediated inhibition of COMP

**Fig. 7:**
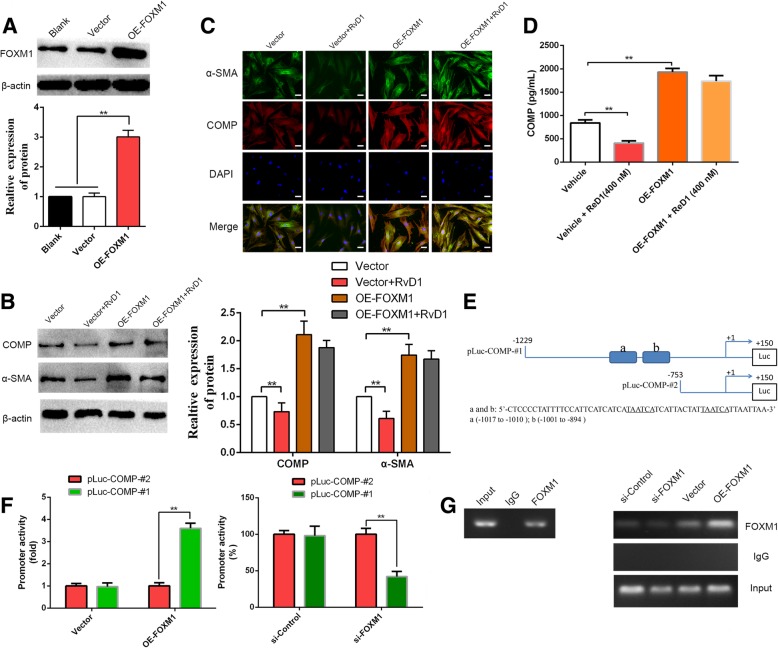
RvD1-mediated inhibits COMP expression through abrogation of FOXM1 recruitment to its promoter. (**a**) pcDNA/FOXM1 could significantly increase FOXM1 expression in CAFs at protein level. n = three independent experiments, ** P < 0.01 by ANOVA. (**b**-c) pcDNA/ Control and pcDNA/ FOXM1 were transfected into CAFs then treated with RvD1, the expression of α-SMA and COMP were examined by western blotting and double immunofluorescence analysis. Magnification is × 400, and scale bars = 20 μm. n = three independent experiments, ** P < 0.01 by ANOVA. (**d**) pcDNA/ Control and pcDNA/ FOXM1 were transfected into CAFs then treated with RvD1, the serection of COMP was measured by Elisa assay. n = three independent experiments, ** P < 0.01 by ANOVA. (**e**) The sequences and positions of putative FOXM1-binding elements on the COMP promoter. (**f**) CAFs were co-transfected with the COMP promoter–luciferase construct pLuc–COMP#1 or pLuc–COMP#2, and 50 nmol/L siFOXM1 or control siRNA, and pcDNA3.1-FOXM1 or pcDNA3.1-vector. Promoter activity was measured by a dual luciferase assay kit. n = three independent experiments, ** P < 0.01 by ANOVA. (**g**) Chromatins were extracted from CAFs, CAFs-siControl, CAFs-siFOXM1, CAFs-vector and CAFs-OE-FOXM1, and the binding of FOXM1 to the COMP promoter was detected by the ChIP assay

**Fig. 8:**
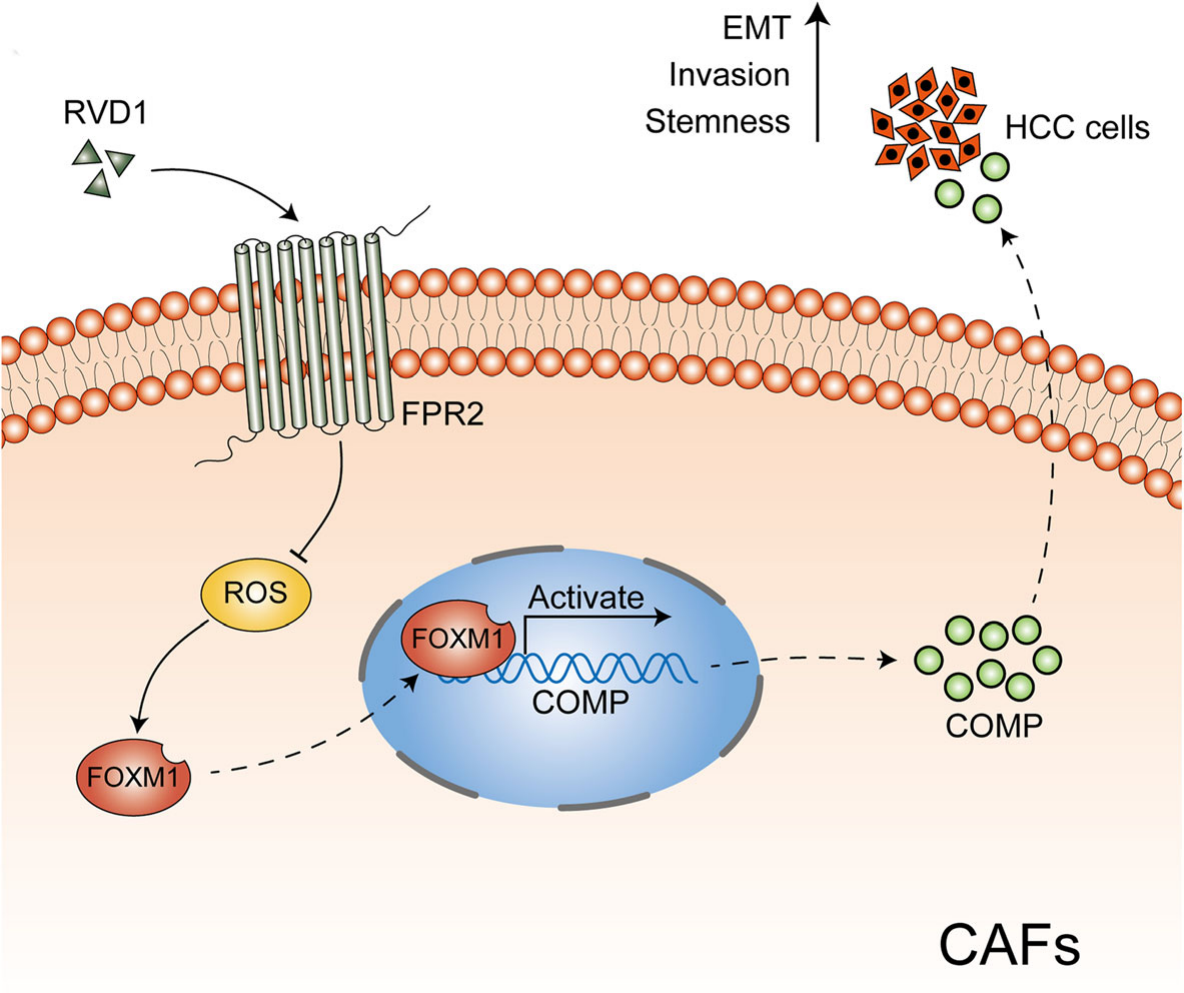
Schematic of the findings of the present study. CAFs-derived COMP induces EMT and stem cell-like phenotypes in HCC cells. However, RvD1, an endogenous proresolving and anti-inflammatory lipid mediator, binds to its receptor-FPR2 on the surface of CAFs, represses ROS mediated FOXM1 binding to the COMP promoter, and then inhibits COMP secretion to block this process

Previous studies have uncovered that FOXM1 exerts its role in a ROS-dependent manner [26]. Thus, we further hypothesized that RvD1 inhibited COMP expression by inhibiting FOXM1. To test this notion, we first overexpressed FOXM1 in CAFs via transfection of a FOXM1 overexpression plasmid (*P* < 0.05, Fig. 7a). RvD1 inhibited the expression and secretion of COMP, which was subsequently rescued by FOXM1 restoration (*P* < 0.05, Fig. 7b-d). Previous studies have reported the sequences of potential FOXM1-binding elements on the promoter: 5-A(C/T)AAA(C/T)AA-3, 5-TAATCA-3, and 5-AGATTGAGTA-3 [41]. Next, to confirm whether FOXM1 could directly regulate the expression of COMP, the binding sites for FOXM1 on the promoter of COMP were analyzed. Interestingly, two potential binding sites for FOXM1 (5-TAATCA-3) were discovered, as shown in Fig. 7e. Next, we generated two luciferase reporter vectors containing COMP promoters: pLuc–COMP-#1 containing two FOXM1-binding sites, and pLuc–COMP-#2 with no potential FOXM1-binding sites (Fig. 7e). The luciferase reporter vectors and FOXM1 overexpression plasmid or siFOXM1 were then co-transfected into CAFs. As shown in Fig. 7f, overexpression of FOXM1 increased whereas knockdown of FOXM1 decreased the COMP promoter activity in the pLuc–COMP-#1 group (*P* < 0.05). However, modulation of FOXM1 expression did not alter the promoter activity in the pLuc–COMP-#2 group (Fig. 7f). To provide evidence that FOXM1 could bind directly to the COMP promoter, we performed a ChIP assay using chromatin extracted from CAFs. As expected, the results indicated that FOXM1 bound directly to the COMP promoter (Fig. 7h), and the modulation of FOXM1 levels impacted the engagement of FOXM1 to COMP promoter (Fig. 7h). These results strongly illustrated the direct binding of FOXM1 to the COMP promoter and regulation of COMP expression at the transcriptional level. In addition, a schematic of the findings of the present study is presented in Fig. 8.

## Discussion

Cancer stem cells require stromal signals to maintain pluripotency and self-renewal capacities to confer successful metastatic colonization [42, 43]. Previous studies have identified stromal fibroblasts as the stem cell niche responsible for the production of periostin, a component of the extracellular matrix, to recruit Wnt1 and Wnt3A and then activate Wnt signaling in breast cancer stem cells. These infiltrating breast cancer cells induce lung fibroblasts expressing periostin to maintain stemness to achieve initial metastasis colonization [44]. Our study also demonstrated that CAFs-derived COMP induced EMT and cancer stem cell-like properties to promote invasion and metastasis of HCC, which was in accord with previous findings that IL-6 secreted by CAFs confers stem-like properties in HCC via the upregulation of stemness-correlated transcription factors including Sox2, Oct4 and Nanog [34]. Thus, educational cancer-associated fibroblasts in the tumor microenvironment play crucial roles in cancer metastasis, and targeting the CAF-provided stem niche may represent a novel strategy for the treatment cancer metastasis.

Most cases of HCC initiate from cirrhotic livers, which contain an abundance of trans-differentiated myofibroblasts from quiescent fibroblasts or hepatic stellate cells [6]. Previous researchers have demonstrated that COMP plays a key role in modulating liver fibrosis through activating MEK1/2 –pERK1/2 signaling and enhancing the synthesis of type 1 collagen in hepatic stellate cells (HSCs), and HSCs-derived COMP facilitates invasion and metastasis of HCC [20]. These results suggest that COMP has an essential role in HCC tumorigenesis initiated in a fibrotic or cirrhotic background. In fact, a positive feedback loop exists between COMP expression and TGF-β signaling [13, 45], in which onset of the fibrotic or cirrhotic process leads to a self-perpetuating cycle with COMP promotion of TGF-β activity and TGF-β facilitation of COMP expression [45, 46]. Previous findings have highlighted that lipoxin A4, another endogenous anti-inflammatory lipid mediator derived from the metabolite of arachidonic acid, can mitigate the invasion and metastasis of pancreatic cancer by inducing inhibition of autocrine TGF-β1 signaling [33]. Interestingly, herein we found that RvD1 inhibited COMP in CAFs in a paracrine manner to modulate EMT and cancer stemness in HCC, providing a promising therapeutic strategy for patients with HCC. However, whether RvD1 inhibits COMP expression by interrupting the positive feedback loop between COMP expression and TGF-β signaling in CAFs requires further clarification.

Our further investigations demonstrated that RvD1 inhibited COMP in CAFs in a paracrine manner via FPR2/ROS/FOXM1 cascades. First, RvD1 suppressed the expression of COMP, FOXM1 and ROS in a receptor-dependent manner; then, manipulation of the ROS level was found to influence the inhibitory efficacy of RvD1 toward the expression of COMP and FOXM1; furthermore, as a critical regulator of oxidative stress, FOXM1 could directly bind to the COMP promoter and regulate COMP expression at the transcriptional level, and abrogation of FOXM1 recruitment through FPR2/ROS signaling was an important mechanism of RvD1-mediated inhibition of COMP expression. These results implicated that oxidative stress-mediated FOXM1 directly modulated COMP expression, and targeting ROS/FOXM1/COMP cascades may provide new avenues to inhibit CAF-induced cancer stemness in HCC.

In this study, we mainly concentrated on the upstream mechanism by which RvD1 provided paracrine inhibition of CAFs-derived COMP via FPR2/ROS/FOXM1 cascades to prevent EMT and cancer stemness in HCC. Nevertheless, further mechanisms by which CAFs-derived COMP regulates stem-like phenotypes in HCC remain unknown. In conjunction with our previous studies [20], we postulated that CAFs-derived COMP may function through binding to its potential receptor on HCC cells and subsequently activating downstream signaling to regulate stem-like properties. It would be of great interest to explore its explicit mechanisms in future studies.

Previous studies have illustrated that conventional chemotherapy, irradiation and targeted therapy are a double-edged sword, as these methods not only result in the killing of cancer cells to reduce tumor burden but also generate tumor-promoting effects via treatment byproducts, i.e., apoptotic tumor cells or tumor cell debris [31]. Moreover, these therapies can trigger a cytokine storm in the tumor stroma, such as the release of IL-6 and TNF-α, as well as activate macrophages to generate pro-inflammatory mediators to stimulate tumor growth and contribute to recurrence [31, 47, 48]. The tumor stroma is also regarded as a niche for the maintenance of cancer stem-like properties and, thus, participates in resistance to conventional therapy [49]. Therefore, overcoming the dilemma between killing tumor cells and stroma resistance is paramount to preventing tumor recurrence after therapy. Intriguingly, RvD1, a novel endogenous anti-inflammatory lipid mediator governing neutrophil influx, macrophage resolution and pro-inflammatory cytokine reduction, promotes the clearance of tumor debris and subsequent inhibition of tumor growth. Furthermore, our study also showed that RvD1 could inhibit CAF-derived COMP in a paracrine manner to suppress EMT and stemness in HCC. Consequently, RvD1, as a novel anti-inflammatory mediator targeting the tumor stroma, may be a useful agent in conjunction with conventional therapy to promote treatment outcomes in HCC patients.

## Conclusions

Our data revealed that RvD1 treatment impeded CAFs-induced cancer stem-like properties and EMT in HCC in vitro under CAFs-HCC cells co-culture conditions. In vivo studies indicated that RvD1 intervention repressed the promoting effects of CAFs on tumor growth and metastasis of HCC. Furthermore, RvD1 inhibited CAFs-induced cancer stemness and EMT in HCC cells by suppressing the secretion of COMP. Mechanistically, RvD1 impaired CAFs-derived COMP in a paracrine manner through targeting FPR2/ROS/FOXM1 signaling to finically abrogate FOXM1 recruitment to the COMP promoter. Taken together, these results support RvD1 as a potential agent to promote treatment outcomes in HCC. In addition, targeting stromal-derived COMP may be an effective strategy to block interactions between the tumor and stroma.

## Additional files

Additional file 1: **Table S1.** A list of the utilized primary antibodies (DOCX 18 kb)

Additional file 2: **Table S2.** Primers sequences for real-time PCR analysis (DOCX 18 kb)

Additional file 3: **Figure S1.** Identification of CAFs derived from HCC patients. (A) CAFs and PTFs were isolated from human HCC tissues and adjacent normal liver tissues. α-SMA expression in CAFs and PTFs were determined by immunofluorescence staining. The magnification of the picture is 400×. Scale bars = 20 μm. (B) qRT-PCR was performed to detect the expression of COMP and α-SMA at mRNA level. n = three independent experiments, **P < 0.01 versus control by t test. (C) Expression levels of Col 1α, fibronectin and α-SMA in CAFs and PTFs were determined by western blotting. * *P* < 0.05 or ** *P* < 0.01versus control by t test. (TIF 806 kb)

Additional file 4: **Figure S2.** RvD1 impeded CAFs-induced EMT and CSC-like properties in HCC cells. (A) Hep3B and SMMC-7721 cells were incubated with CM from CAFs (CM_CAFs_) or CM from CAFs pre-treated with RvD1(400 nM) (CM_CAFs + RvD1_) for 48 h, the relative expression of stemness markers (OCT4, Nanog, Sox2), and epithelial-mesenchymal transition markers (E-cadherin, N-cadherin, vimentin) at protein level were analyzed and plotted. n = three independent experiments, * *P* < 0.05 or ** *P* < 0.01 by ANOVA. (B and C) Hep3B and SMMC-7721 cells were treated with CMCAFs and CMCAFs+RvD1 for 48 h, and western blotting analysis was performed to test the expression of other stemness markers (CD44, EPCAM, CD90). n = three independent experiments, * *P* < 0.05 or ** *P* < 0.01 by ANOVA. (TIF 1009 kb)

Additional file 5: **Figure S3.** The content of RvD1 in HCC tissues was significantly decreased compared with the adjacent non-tumor samples. (A) The content of RvD1 in HCC and the adjacent non-tumor tissues was examined by an Elisa kit. n = three independent experiments, **P < 0.01 versus control by t test. (B) The interaction of 15-LOX with 5-LOX participates in the synthetic process of DHA-derived resolvins. (C) The expression of 15-LOX in HCC and the adjacent non-tumor tissues was determined by western blotting analysis. n = three independent experiments, * P < 0.05 or **P < 0.01 versus control by t test. (TIF 5476 kb)

Additional file 6: **Figure S4.** RvD1 harbored no obvious effects on tumor cells. (A) Hep3B and SMMC-7721 cells were treated with RvD1 (0, 200, 400 and 800 nM) for 72 h, then, the cell viability was assessed by MTT assay. (B) Hep3B and SMMC-7721 cells were intervened with RvD1 (400 nM) 24 h, then Transwell invasion assay was performed to evaluate the invasive capability of HCC cells. (TIF 3742 kb)

Additional file 7: **Figure S5.** RvD1 repressed the expression of COMP and the nuclear localization of FOXM1. (A) The effects of RvD1 on the nuclear localization of FOXM1 were detected by immunofluorescence analysis. (B) Double immunofluorescence staining was used to examine the effects of RvD1 on the expression of α-SMA and COMP. The magnification of the picture is 400×. Scale bars = 20 μm. (TIF 1377 kb)

Additional file 8: **Figure S6.** RvD1 inhibits the expression of F-actin in a HCC-CAFs direct co-culture model. HCC cells and CAFs were cultured together in the presence or absence of RvD1 (400 nM) for 48 h. Then, immunofluorescence staining was performed to evaluate F-actin expression in these cells. Magnification is × 400, and scale bars = 20 μm. (TIF 341 kb)

Additional file 9: **Figure S7.** RvD1 inhibited CAFs-induced EMT and CSC-like properties in HCC cells via targeting paracrine of COMP. (A) The relative expression of CSC and EMT markers at protein level was analyzed and plotted after CM_CAFs_, CM_CAFs + RvD1_, CM_CAFs_ + anti-COMP and CM_CAFs + RvD1_ + rh-COMP treatments. n = three independent experiments, * *P* < 0.05 or ** P < 0.01 by ANOVA. (B) Hep3B and SMMC-7721 cells were incubated with CM_CAFs_, CM_CAFs + RvD1_, CM_CAFs_ + anti-COMP and CM_CAFs + RvD1_ + rh-COMP for 24, 48, 72 and 96 h, and cell viability were assessed by MTT assay. * P < 0.05, ** *P* < 0.01. n = three independent experiments, * *P* < 0.05 or ** P < 0.01 by ANOVA. (C and D) After treated with CM_CAFs_, CM_CAFs + RvD1_, CM_CAFs_ + anti-COMP and CM_CAFs + RvD1_ + rh-COMP, other CSC markers (CD44, EPCAM, CD90) were determined by western blotting. n = three independent experiments, * P < 0.05 or ** P < 0.01 by ANOVA. (TIF 1543 kb)

Additional file 10: **Figure S8.** Silencing ALX/FPR2 can reverse the efficacy of RvD1 on the expression of COMP and nuclear localization of FOXM1. (A) CAFs were transfected with siRNA targeting ALX/FPR2 (si-FPR2) or negative control (si-NC), and 24 h later, 400 nM RvD1 or vehicle were utilized to treat these cells for 48 h. Subsequently, double immunofluorescence analysis was used to detect ALX/FPR2 and COMP. (B) The nuclear localization of FOXM1 in CAFs administered as above description. The magnification is 400×. Scale bars = 20 μm. (TIF 1710 kb)

Additional file 11: **Figure S9.** Manipulation of ROS level revered the effects of RvD1 on the expression of COMP and nuclear localization of FOXM1. (A) CAFs were treated with RvD1, NAC, RvD1 + H_2_O_2_, and H_2_O_2_ for 48 h, α-SMA and COMP expression in CAFs were determined by double immunofluorescence staining. The magnification is 400×, and the scale bars = 20 μm. (B) The nuclear localization of FOXM1 in CAFs after manipulation of ROS level was detected by immunofluorescence staining. The magnification is 400×, and the scale bars = 20 μm. (TIF 2221 kb)

## Abbreviations

ALX/FPR2: Lipoxin A4 receptor/formyl pep-tide receptor 2 (ALX/FPR2)
anti-COMP: Human COMP neutralization antibody
CAFs: Cancer-associated fibroblasts
CCL2: Chemokine (C-C motif) ligand 2
CM: Conditioned medium
COMP: Cartilage oligomeric matrix protein
CSC: Cancer stem cell
CXCL1: Chemokine (C-X-C motif) ligand 1
CXCL8: Chemokine (C-X-C motif) ligand 8
DHA: Docose hexaenoie acid
EGF: Epidermal growth factor
EMT: Epithelial-mesenchymal transition
EPA: Eicosapntemacnioc acid
FGF: Fibroblast growth factor
FOXM1: Forkhead box protein M1
HAS2: Hyaluronan synthase 2
HCC: Hepatocellular carcinoma
HGF: Hepatocyte growth factor
HSCs: Hepatic stellate cells
IL-6: Interleukin-6
IL-8: Interleukin-8
LOX: Lipoxygenase
MMP2: Matrix metalloproteinase 2
NAC: N-acetyl-L-cysteine
PTFs: Peritumoral fibroblasts
rh-COMP: Recombinant human COMP protein
ROS: Reactive oxygen species
RvD1: Resolvin D1
SCGF-β: Stem cell growth factor-β
TGF-β: Transforming growth factor-β
TME: Tumor microenvironment
TNF-α: Tumor necrosis factor-α
α-SMA: α-smooth muscle
