# Supplementary material for: Resolvin D1 prevents epithelial-mesenchymal transition and reduces the stemness features of hepatocellular carcinoma by inhibiting paracrine of cancer-associated fibroblast-derived COMP
Source: J Exp Clin Cancer Res. 2019 Apr 18;38:170. doi: 10.1186/s13046-019-1163-6 (PMC6472102; doi:10.1186/s13046-019-1163-6)
Supplement: Supplementary file 1 — Table S1. A list of the utilized primary antibodies (DOCX 18 kb) [file 13046_2019_1163_MOESM1_ESM.docx]

**Table S1.** A list of the utilized primary antibodies

| **Antibody** | **Dilution & Use** | **Company** |
| --- | --- | --- |
| Rabbit anti-E-cadherin | 1:1000 (WB) | Cell Signaling Technology |
| Rabbit anti-N-cadherin | 1:1000 (WB) | Cell Signaling Technology |
| Rabbit anti-vimentin | 1:1000 (WB) | Cell Signaling Technology |
| Rabbit anti-MMP2 | 1:500 (WB) | Cell Signaling Technology |
| Rabbit anti-EPCAM | 1:1000 (WB) | Cell Signaling Technology |
| Rabbit anti-Sox2 | 1:1000 (WB) | Abcam |
| Rabbit anti-OCT4 | 1:1000 (WB) | Abcam |
| Rabbit anti-Nanog | 1:1000 (WB) | Abcam |
| Rabbit anti-ki-67 | 1:500 (IHC) | Abcam |
| Rabbit anti-CD44 | 1:1000 (WB) | Abcam |
| Rabbit anti-CD90 | 1:2000 (WB) | Abcam |
| Rabbit anti-15 Lipoxygenase 1 | 1:500 (WB) | Abcam |
| Rabbit anti-COMP | 1:1000 (WB),1:200 (IF) | Abcam |
| Mouse anti-β-actin | 1:10,000 (WB) | Sigma |
| Mouse anti-FOXM1 | 1:1000 (WB),1:200 (IF) | Santa Cruz Biotechnology |
| Rabbit anti-Collgea-1α | 1:1000 (WB) | Cell Signaling Technology |
| Mice anti-α-SMA | 1:1000(WB),1:500 (IF) | Abcam |
| Mice anti-HAS2 | 1:2000 (WB) | Abcam |
| Rabbit anti-CTGF | 1:1000 (WB) | Abcam |
| Rabbit anti-ALX/FPR2 | 1:600 (WB) | Abcam |
| Rabbit anti-GPR32 | 1:1000 (WB) | Abcam |
| Goat anti-rabbit IgG-HRP | 1:10,000 (WB) | Abbkine. Inc |
| Goat anti-mouse IgG-HRP | 1:10,000 (WB) | Abbkine. Inc |
| Goat anti-mouse FITC IgG antibody | 1:150 (IF) | Abbkine. Inc |
| Goat anti-Rabbit dylight 594  (red) IgG antibody | 1:150 (IF) | Abbkine. Inc |
| Goat anti-mouse dylight 594  (red) IgG antibody | 1:150 (IF) | Abbkine. Inc |
