# Supplementary material for: Resolvin D1 prevents epithelial-mesenchymal transition and reduces the stemness features of hepatocellular carcinoma by inhibiting paracrine of cancer-associated fibroblast-derived COMP
Source: J Exp Clin Cancer Res. 2019 Apr 18;38:170. doi: 10.1186/s13046-019-1163-6 (PMC6472102; doi:10.1186/s13046-019-1163-6)
Supplement: Supplementary file 2 — Table S2. Primers sequences for real-time PCR analysis (DOCX 18 kb) [file 13046_2019_1163_MOESM2_ESM.docx]

**Table S2.** Primers sequences for real-time PCR analysis

| **Gene** | **Primer Sequences** |
| --- | --- |
| α-SMA | Forward: 5′-CCCCATCTATGAGGGCTATG-3′ |
|  | Reverse: 5′-CAGTGGCCATCTCATTTTCA-3′ |
| COMP | Forward: 5′- CGACTATGCGGGCTTCATCT-3′ |
|  | Reverse: 5′- TGCCAAACCTGAGTTCCAGT -3′ |
| COMP  for CHIP | Forward: 5′- TGAGAGTCCCAGGTCTCAACT-3′  Reverse: 5′- TTTGCTCCATCTGCTTCCAC -3′ |
| Col 1α1 | Forward: 5′-GTACTGGATTGACCCCAACC-3′ |
|  | Reverse: 5′-CGCCATACTCGAACTGGAAT-3′ |
| Fibronectin | Forward: 5′-CAGTGGGAGACCTCGAGAAG-3′  Reverse: 5′-TCCCTCGGAACATCAGAAAC-3′ |
| Col 3α1 | Forward: 5′-AAGAAGGCCCTGAAGCTGAT-3′ |
|  | Reverse: 5′-GTGTTTCGTGCAACCATCCT-3′ |
| HAS2 | Forward: 5′-GGGACGAAGTGTGGATTATGT-3′  Reverse: 5′-GAGATCCAGGAATCGTACTTGTT-3′ |
| CTGF | Forward: 5′-CTTTGGCCCAGACCCAACTA-3′  Reverse: 5′-GGCTCTGCTTCTCTAGCCTG-3′ |
| MMP2 | Forward: 5′-AGGAGGAGAAGGCTGTGTTC-3′  Reverse: 5′-CTCCAGTTAAAGGCGGCATC-3′ |
| FOXM1 | Forward: 5′-ACGTCCCCAAGCCAGGCTC-3′  Reverse: 5′-CTACTGTAGCTCAGGAATAA-3′ |
| GAPDH | Forward: 5′-ACCACAGTCCATGCCATCAC-3′ |
|  | Reverse: 5′-TCCACCACCCTGTTGCTGAT-3′ |
