# Supplementary figures and images for: Resolvin D1 prevents epithelial-mesenchymal transition and reduces the stemness features of hepatocellular carcinoma by inhibiting paracrine of cancer-associated fibroblast-derived COMP
Source: J Exp Clin Cancer Res. 2019 Apr 18;38:170. doi: 10.1186/s13046-019-1163-6 (PMC6472102; doi:10.1186/s13046-019-1163-6)

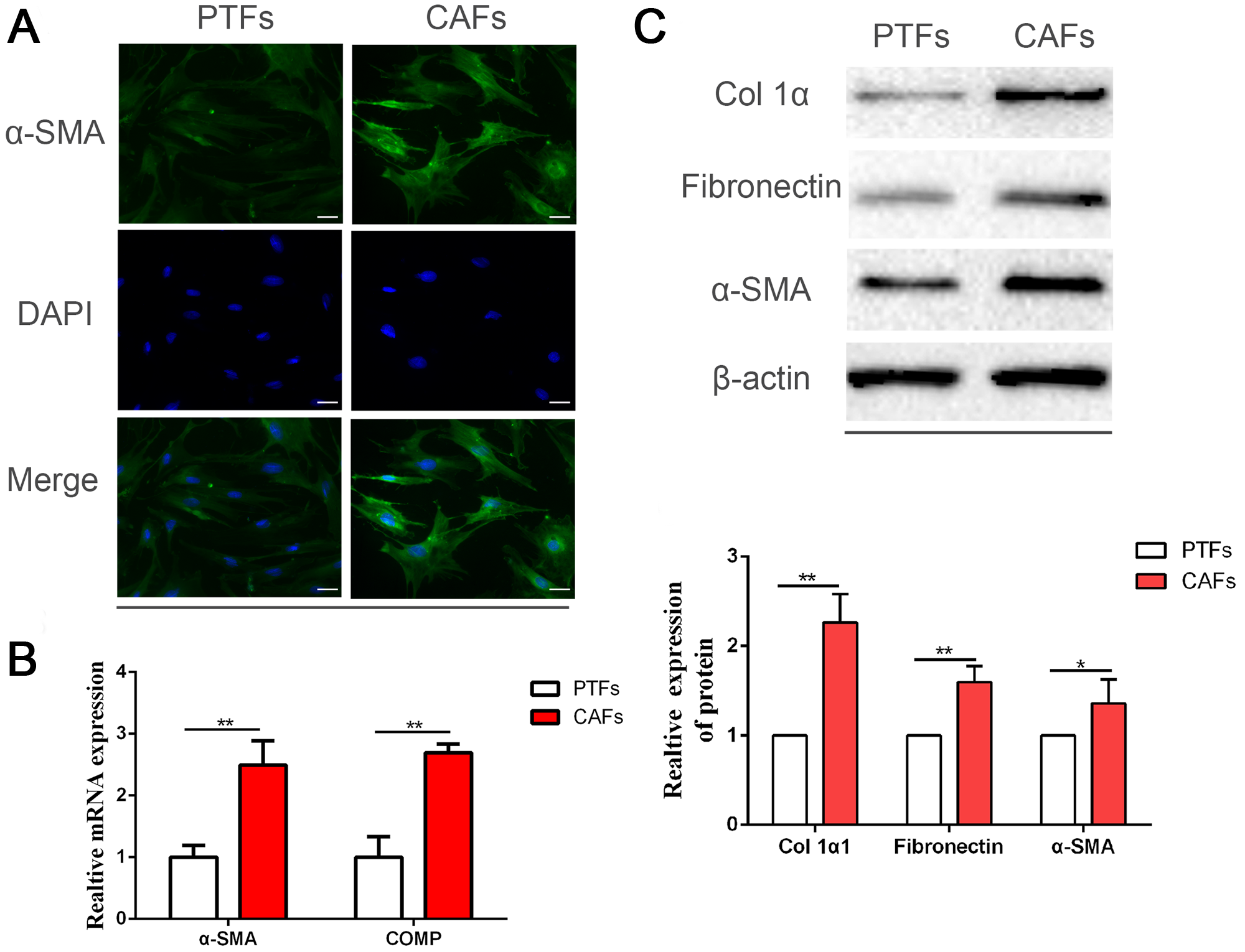

Supplement: Supplementary file 3 — Figure S1. Identification of CAFs derived from HCC patients. (A) CAFs and PTFs were isolated from human HCC tissues and adjacent normal liver tissues. α-SMA expression in CAFs and PTFs were determined by immunofluorescence staining. The magnification of the picture is 400×. Scale bars = 20 μm. (B) qRT-PCR was performed to detect the expression of COMP and α-SMA at mRNA level. n = three independent experiments, **P < 0.01 versus control by t test. (C) Expression levels of Col 1α, fibronectin and α-SMA in CAFs and PTFs were determined by western blotting. * P < 0.05 or ** P < 0.01versus control by t test. (TIF 806 kb) [file 13046_2019_1163_MOESM3_ESM.tif]

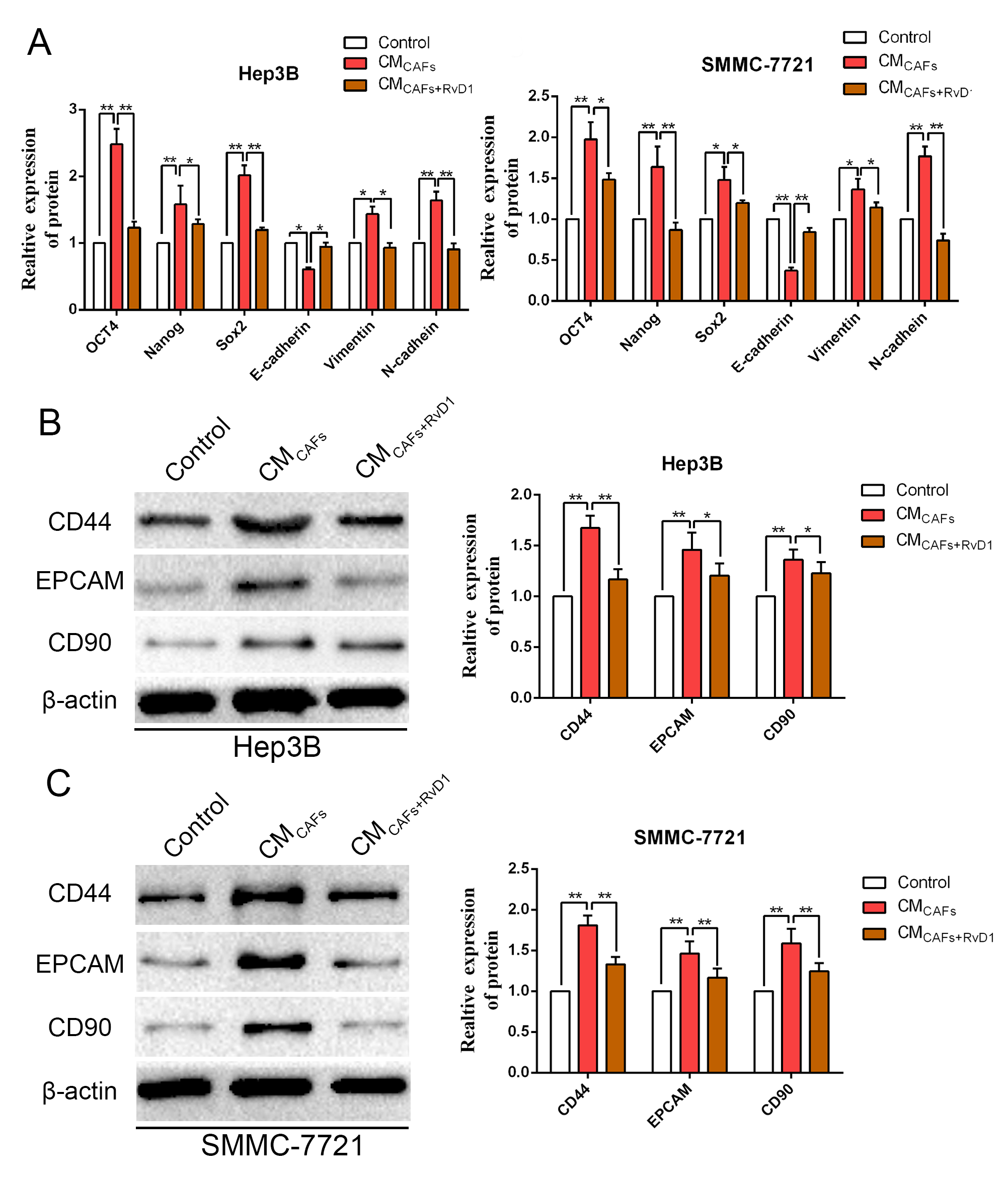

Supplement: Supplementary file 4 — Figure S2. RvD1 impeded CAFs-induced EMT and CSC-like properties in HCC cells. (A) Hep3B and SMMC-7721 cells were incubated with CM from CAFs (CMCAFs) or CM from CAFs pre-treated with RvD1(400 nM) (CMCAFs + RvD1) for 48 h, the relative expression of stemness markers (OCT4, Nanog, Sox2), and epithelial-mesenchymal transition markers (E-cadherin, N-cadherin, vimentin) at protein level were analyzed and plotted. n = three independent experiments, * P < 0.05 or ** P < 0.01 by ANOVA. (B and C) Hep3B and SMMC-7721 cells were treated with CMCAFs and CMCAFs+RvD1 for 48 h, and western blotting analysis was performed to test the expression of other stemness markers (CD44, EPCAM, CD90). n = three independent experiments, * P < 0.05 or ** P < 0.01 by ANOVA. (TIF 1009 kb) [file 13046_2019_1163_MOESM4_ESM.tif]

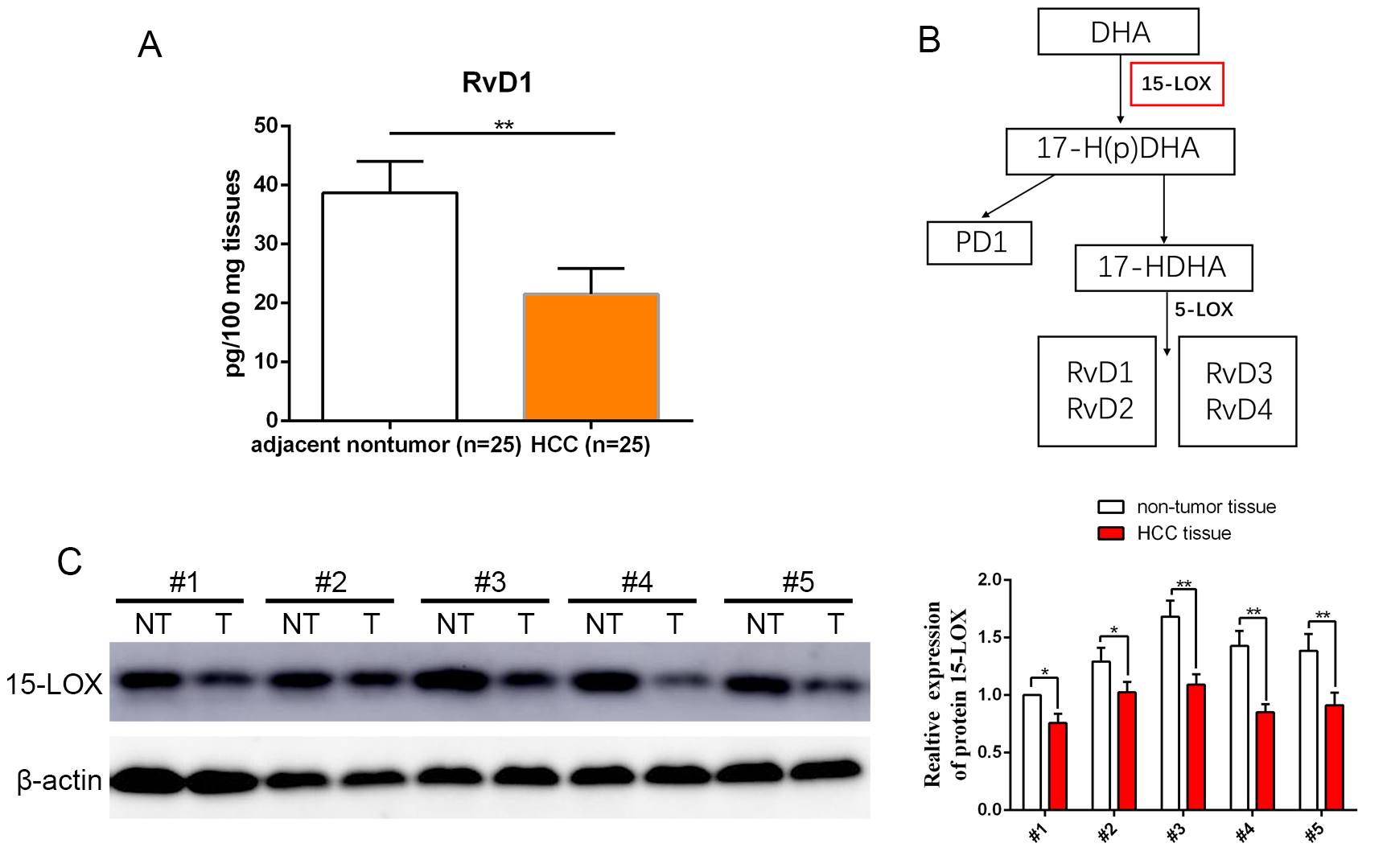

Supplement: Supplementary file 5 — Figure S3. The content of RvD1 in HCC tissues was significantly decreased compared with the adjacent non-tumor samples. (A) The content of RvD1 in HCC and the adjacent non-tumor tissues was examined by an Elisa kit. n = three independent experiments, **P < 0.01 versus control by t test. (B) The interaction of 15-LOX with 5-LOX participates in the synthetic process of DHA-derived resolvins. (C) The expression of 15-LOX in HCC and the adjacent non-tumor tissues was determined by western blotting analysis. n = three independent experiments, * P < 0.05 or **P < 0.01 versus control by t test. (TIF 5476 kb) [file 13046_2019_1163_MOESM5_ESM.tif]

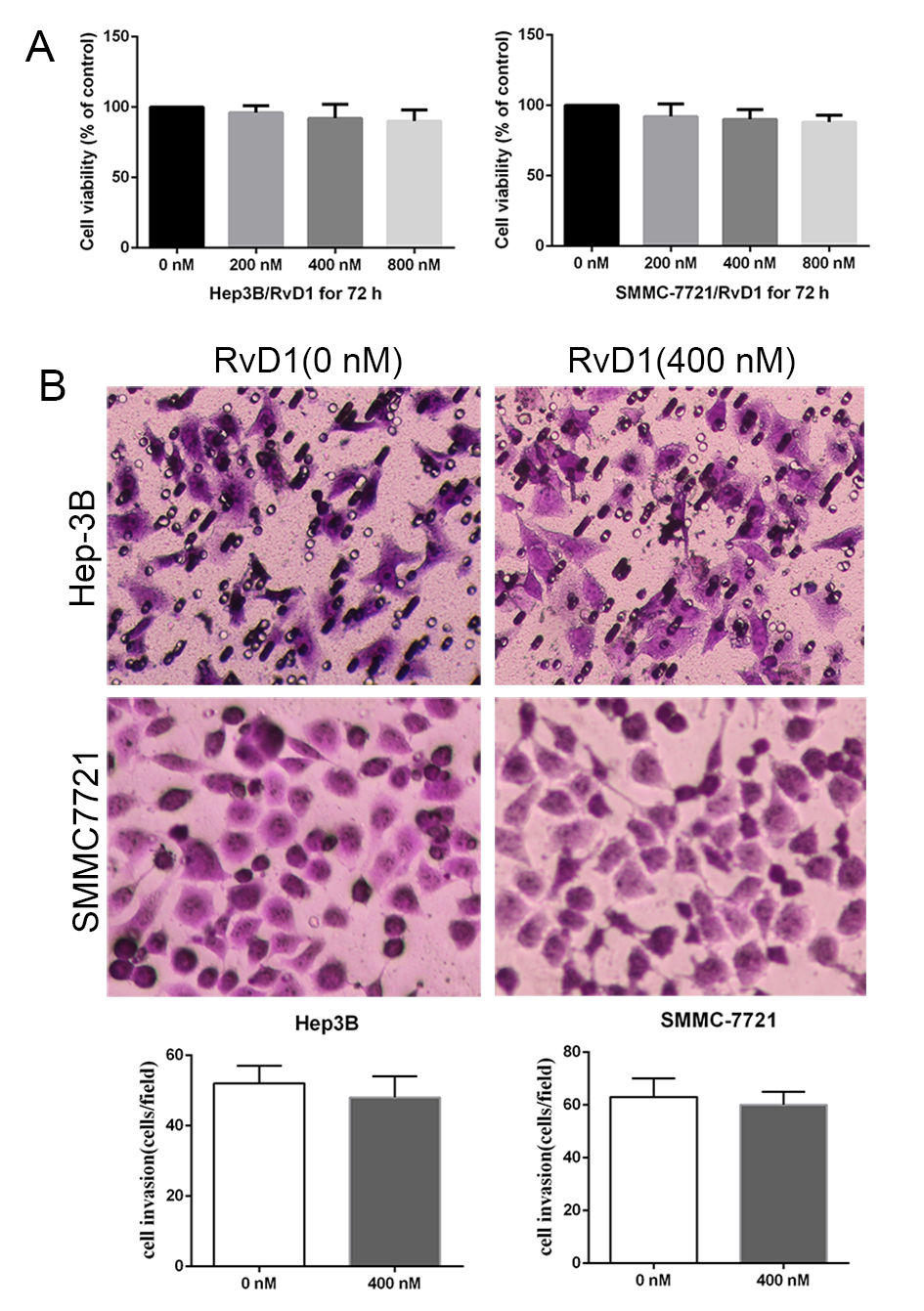

Supplement: Supplementary file 6 — Figure S4. RvD1 harbored no obvious effects on tumor cells. (A) Hep3B and SMMC-7721 cells were treated with RvD1 (0, 200, 400 and 800 nM) for 72 h, then, the cell viability was assessed by MTT assay. (B) Hep3B and SMMC-7721 cells were intervened with RvD1 (400 nM) 24 h, then Transwell invasion assay was performed to evaluate the invasive capability of HCC cells. (TIF 3742 kb) [file 13046_2019_1163_MOESM6_ESM.tif]

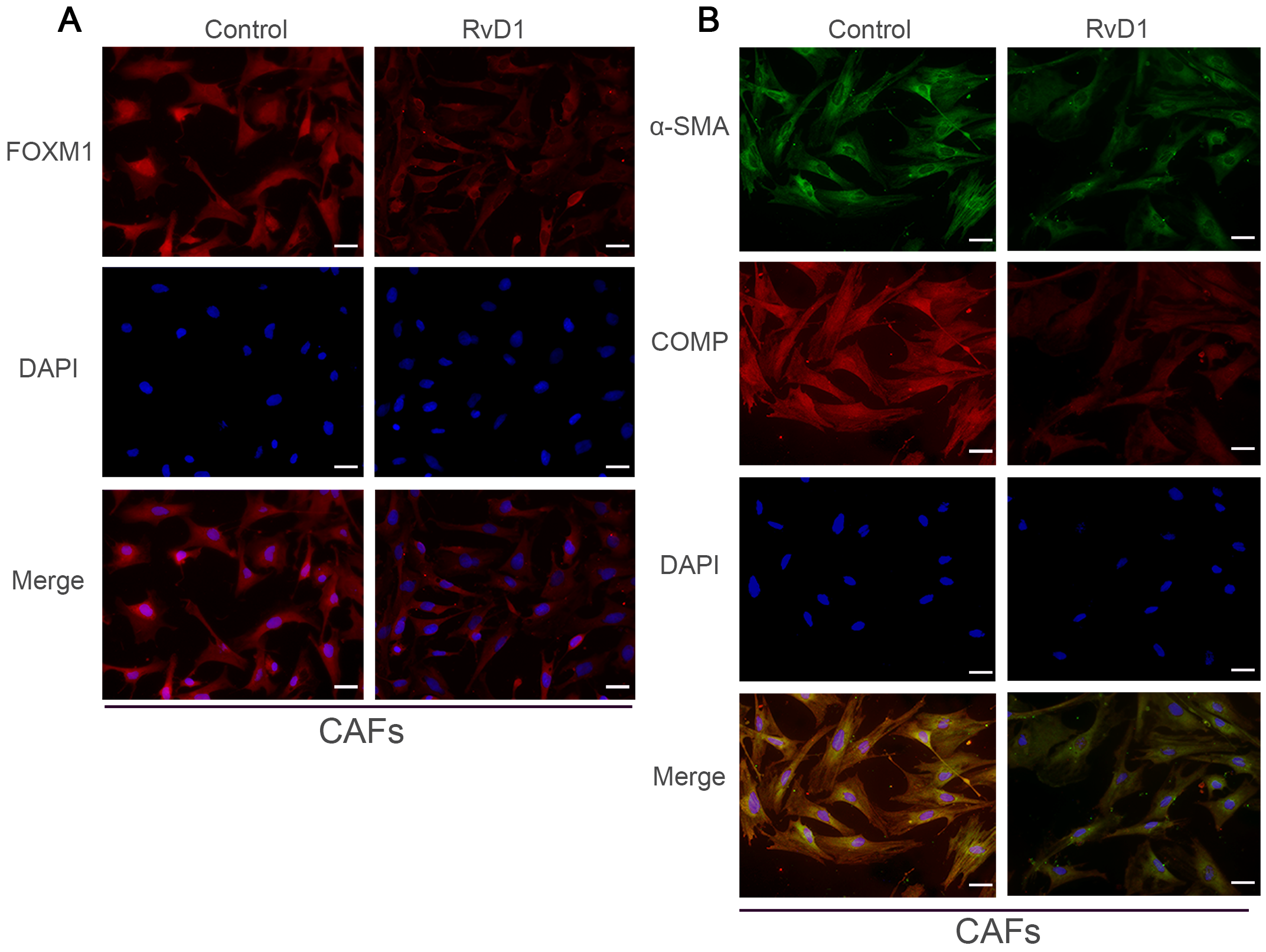

Supplement: Supplementary file 7 — Figure S5. RvD1 repressed the expression of COMP and the nuclear localization of FOXM1. (A) The effects of RvD1 on the nuclear localization of FOXM1 were detected by immunofluorescence analysis. (B) Double immunofluorescence staining was used to examine the effects of RvD1 on the expression of α-SMA and COMP. The magnification of the picture is 400×. Scale bars = 20 μm. (TIF 1377 kb) [file 13046_2019_1163_MOESM7_ESM.tif]

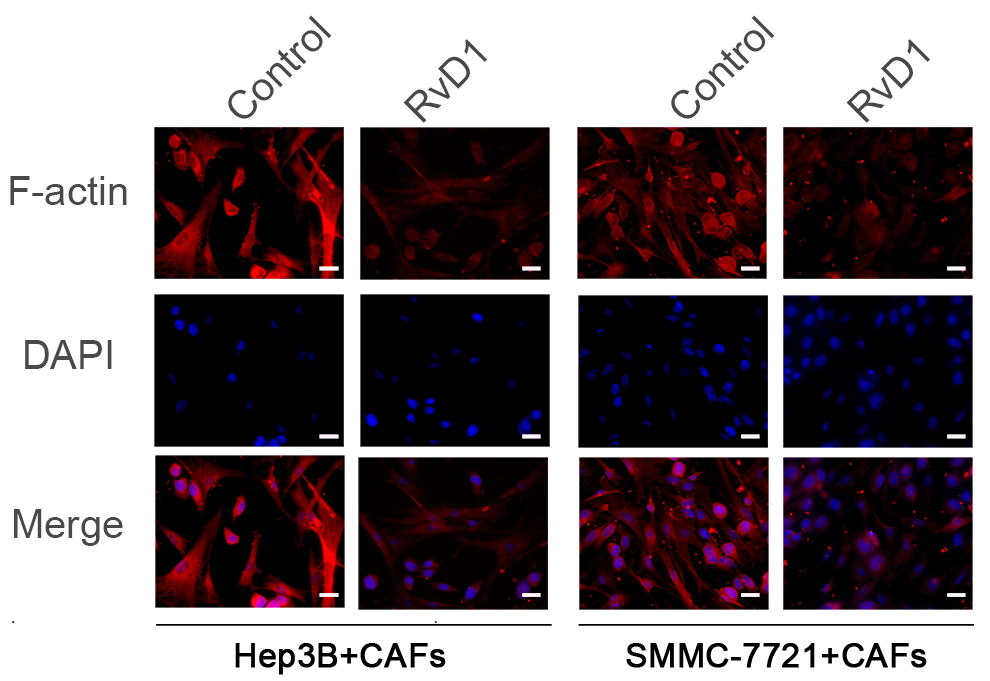

Supplement: Supplementary file 8 — Figure S6. RvD1 inhibits the expression of F-actin in a HCC-CAFs direct co-culture model. HCC cells and CAFs were cultured together in the presence or absence of RvD1 (400 nM) for 48 h. Then, immunofluorescence staining was performed to evaluate F-actin expression in these cells. Magnification is × 400, and scale bars = 20 μm. (TIF 341 kb) [file 13046_2019_1163_MOESM8_ESM.tif]

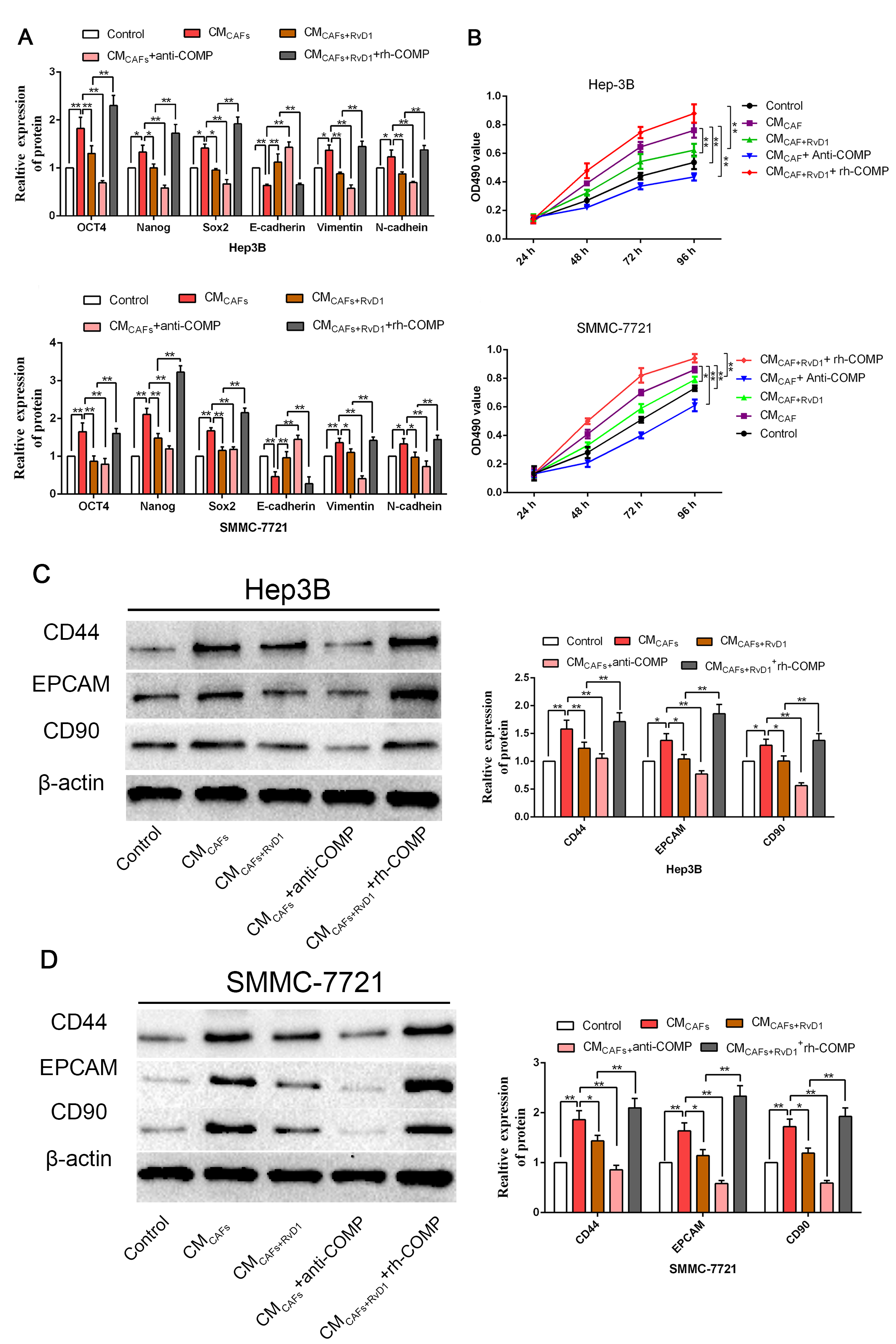

Supplement: Supplementary file 9 — Figure S7. RvD1 inhibited CAFs-induced EMT and CSC-like properties in HCC cells via targeting paracrine of COMP. (A) The relative expression of CSC and EMT markers at protein level was analyzed and plotted after CMCAFs, CMCAFs + RvD1, CMCAFs + anti-COMP and CMCAFs + RvD1 + rh-COMP treatments. n = three independent experiments, * P < 0.05 or ** P < 0.01 by ANOVA. (B) Hep3B and SMMC-7721 cells were incubated with CMCAFs, CMCAFs + RvD1, CMCAFs + anti-COMP and CMCAFs + RvD1 + rh-COMP for 24, 48, 72 and 96 h, and cell viability were assessed by MTT assay. * P < 0.05, ** P < 0.01. n = three independent experiments, * P < 0.05 or ** P < 0.01 by ANOVA. (C and D) After treated with CMCAFs, CMCAFs + RvD1, CMCAFs + anti-COMP and CMCAFs + RvD1 + rh-COMP, other CSC markers (CD44, EPCAM, CD90) were determined by western blotting. n = three independent experiments, * P < 0.05 or ** P < 0.01 by ANOVA. (TIF 1543 kb) [file 13046_2019_1163_MOESM9_ESM.tif]

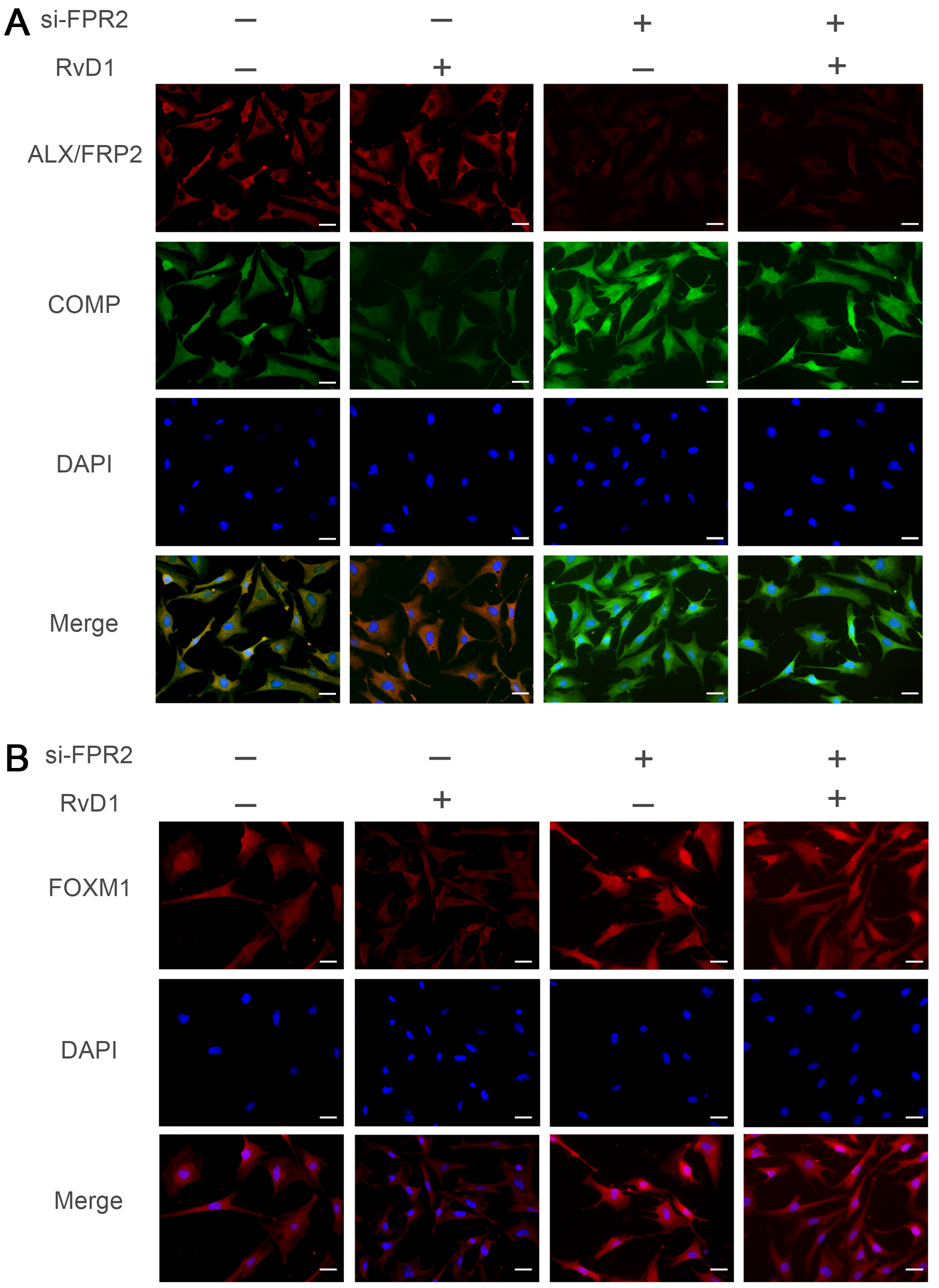

Supplement: Supplementary file 10 — Figure S8. Silencing ALX/FPR2 can reverse the efficacy of RvD1 on the expression of COMP and nuclear localization of FOXM1. (A) CAFs were transfected with siRNA targeting ALX/FPR2 (si-FPR2) or negative control (si-NC), and 24 h later, 400 nM RvD1 or vehicle were utilized to treat these cells for 48 h. Subsequently, double immunofluorescence analysis was used to detect ALX/FPR2 and COMP. (B) The nuclear localization of FOXM1 in CAFs administered as above description. The magnification is 400×. Scale bars = 20 μm. (TIF 1710 kb) [file 13046_2019_1163_MOESM10_ESM.tif]

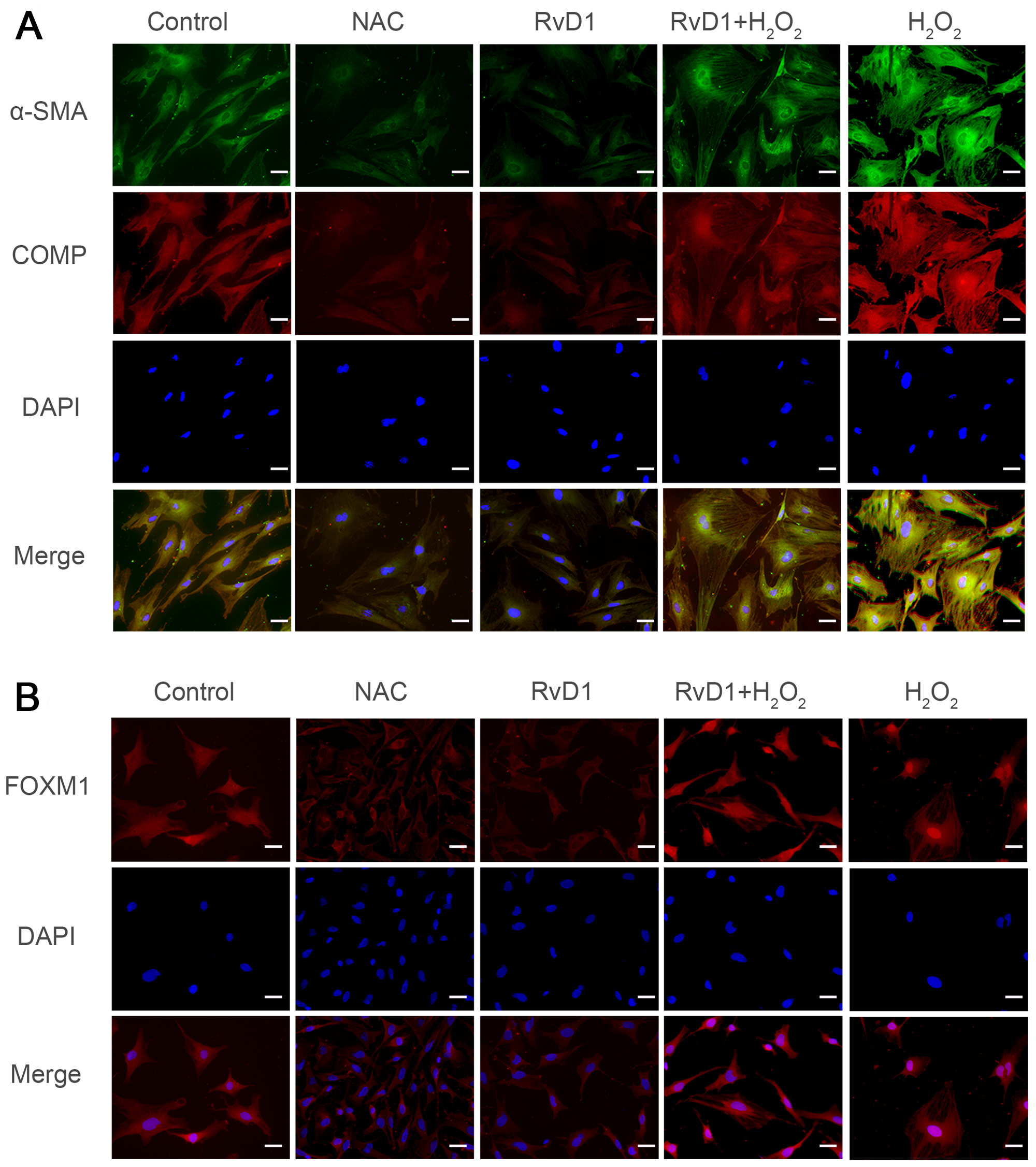

Supplement: Supplementary file 11 — Figure S9. Manipulation of ROS level revered the effects of RvD1 on the expression of COMP and nuclear localization of FOXM1. (A) CAFs were treated with RvD1, NAC, RvD1 + H2O2, and H2O2 for 48 h, α-SMA and COMP expression in CAFs were determined by double immunofluorescence staining. The magnification is 400×, and the scale bars = 20 μm. (B) The nuclear localization of FOXM1 in CAFs after manipulation of ROS level was detected by immunofluorescence staining. The magnification is 400×, and the scale bars = 20 μm. (TIF 2221 kb) [file 13046_2019_1163_MOESM11_ESM.tif]
